# The penile microbiota of Black South African men: relationship with human papillomavirus and HIV infection

**DOI:** 10.1186/s12866-020-01759-x

**Published:** 2020-04-06

**Authors:** Harris Onywera, Anna-Lise Williamson, Luca Cozzuto, Sarah Bonnin, Zizipho Z. A. Mbulawa, David Coetzee, Julia Ponomarenko, Tracy L. Meiring

**Affiliations:** 1 Institute of Infectious Disease and Molecular Medicine, University of Cape Town, Cape Town, South Africa; 2 Division of Medical Virology, Department of Pathology, Faculty of Health Sciences, University of Cape Town, Cape Town, South Africa; 3 Center for Genomic Regulation (CRG), The Barcelona Institute of Science and Technology, Barcelona, Spain; 4 SAMRC Gynaecological Cancer Research Centre, University of Cape Town, Cape Town, South Africa; 5 Department of Laboratory Medicine and Pathology, National Health Laboratory Service and Walter Sisulu University, Nelson Mandela Academic Hospital, Fort Gale, Mthatha, South Africa; 6 Center for Infectious Disease Epidemiology and Research, School of Public Health and Family Medicine, University of Cape Town, Cape Town, South Africa; 7 University of Pompeu Fabra, Barcelona, Spain

**Keywords:** Microbiota, Penile, Human papillomavirus (HPV), HIV

## Abstract

**Background:**

To date, the microbiota of the human penis has been studied mostly in connection with circumcision, HIV risk and female partner bacterial vaginosis (BV). These studies have shown that male circumcision reduces penile anaerobic bacteria, that greater abundance of penile anaerobic bacteria is correlated with increased cytokine levels and greater risk of HIV infection, and that the penile microbiota is an important harbour for BV-associated bacteria. While circumcision has been shown to significantly reduce the risk of acquiring human papillomavirus (HPV) infection, the relationship of the penile microbiota with HPV is still unknown. In this study, we examined the penile microbiota of HPV-infected men as well as the impact of HIV status.

**Results:**

The penile skin microbiota of 238 men from Cape Town (South Africa) were profiled using Illumina sequencing of the V3-V4 hypervariable regions of the 16S rRNA gene. *Corynebacterium* and *Prevotella* were found to be the most abundant genera. Six distinct community state types (CSTs) were identified. CST-1, dominated by *Corynebacterium*, corresponded to less infections with high-risk HPV (HR-HPV) relative to CSTs 2–6. Men in CST-5 had greater relative abundances of *Prevotella*, *Clostridiales*, and *Porphyromonas* and a lower relative abundance of *Corynebacterium*. Moreover, they were significantly more likely to have HPV or HR-HPV infections than men in CST-1. Using a machine learning approach, we identified greater relative abundances of the anaerobic BV-associated bacteria (*Prevotella*, *Peptinophilus*, and *Dialister*) and lower relative abundance of *Corynebacterium* in HR-HPV-infected men compared to HR-HPV-uninfected men. No association was observed between HIV and CST, although the penile microbiota of HIV-infected men had greater relative abundances of *Staphylococcus* compared to HIV-uninfected men.

**Conclusions:**

We found significant differences in the penile microbiota composition of men with and without HPV and HIV infections. HIV and HR-HPV infections were strongly associated with greater relative abundances of *Staphylococcus* and BV-associated bacterial taxa (notably *Prevotella*, *Peptinophilus* and *Dialister*), respectively*.* It is possible that these taxa could increase susceptibility to HIV and HR-HPV acquisition, in addition to creating conditions in which infections persist. Further longitudinal studies are required to establish causal relationships and to determine the extent of the effect.

## Background

To date over 40 human papillomavirus (HPV) genotypes have been shown to infect the anogenital tract, with 13 of these genotypes classified as oncogenic or high-risk HPV (HR-HPV) by the World Health Organisation [1]. Persistent infection with HR-HPVs is causally associated with the development of cervical, vulvar and vaginal cancers in women, penile cancer in men, as well as anal and oropharyngeal cancers in both sexes [1]. Human immunodeficiency virus (HIV)-infected men have a significantly higher incidence, prevalence and persistence of HPV [2, 3]. The burden of HPV-related dysplasias is significantly greater in HIV-infected than HIV-uninfected individuals (reviewed in [4]).

The best evidence to date for a potential role of the penile microbiome in sexually transmitted infections (STIs) and HIV acquisition have come from studies examining medical circumcision [5, 6]. Male circumcision reduces the risk of HPV and HIV infection in men [7–11]. Male circumcision has been found to alter the penile microbiome by significantly reducing bacterial diversity and load [12, 13]. Pathogenic bacteria and dysbiosis in the penile microbiota, characterised by the presence of bacterial vaginosis (BV)-associated anaerobic bacteria such as *Prevotella,* have been identified as a key risk factor for HIV acquisition in uncircumcised men [5, 14]. Several recent studies [15–22] have indicated that specific genital bacteria, which are more prevalent or abundant in uncircumcised men, could stimulate local immune responses that enhance epithelial inflammation and HIV target cell recruitment. This suggests that HIV acquisition could be linked to proinflammatory anaerobic bacteria in the penile bacterial microbiota.

At present, there is no data on the impact of HIV infection on penile microbiota. Moreover, the role of the penile microbiota in HPV infection in men is mostly unknown. To fill this gap, we report here a study of penile skin microbiota in 238 men from the HPV Couples Cohort Study in South Africa [2].

## Results

### Study subjects

This cross-sectional study is a retrospective analysis of samples collected during a 2-year longitudinal HPV Couples Cohort Study as detailed elsewhere [2]. The parent study was designed to investigate genital HPV prevalence and sharing among heterosexually-active Black South African HIV-concordant and -discordant couples. Penile specimens were collected from heterosexually-active Xhosa-speaking adult men aged 19–67 years recruited from Gugulethu, Cape Town, South Africa. The baseline characteristics of the 238 South African men included in the study are summarised in Table 1.

**Table 1:** Baseline characteristics of the 238 heterosexually-active Black South African men

**Characteristic**	**Participants (*N*** **= 238)**
Age (years)^a^	36.0 (30.0–44.0)
Age at sexual debut (years)^ab^	17.0 (15.0–18.0)
Number of lifetime sexual partners	6.0 (3.0–13.0)
Number of sexual acts with study partner in last month^ab^	4.0 (2.0–10.0)
Current use of condom (% (n/N))	64.1 (141/220)
HPV-positive (% (n/N))	54.6 (130/238)
HR-HPV-positive (% (n/N))	42.9 (102/238)
HIV-positive (% (n/N))	37.0 (88/238)
CD4^+^ T-cell count if HIV-positive (cells/μl)^ab^	334 (232–478)
HIV viral load if HIV-positive (copies/ml)^ab^	4.1 log_10_ (3.7–4.7)
Circumcised (% (n/N))	94.3 (215/228)
Cigarette use (% (n/N))	
Never smoked	16.9 (40/237)
Ex-smoker	16.9 (40/237)
Current smoker	66.2 (157/237)

Abbreviations: *HPV* Human papillomavirus, *HR-HPV* High-risk human papillomavirus, *HIV* Human immunodeficiency virus

^a^Continuous variables are expressed as medians with interquartile ranges (IQRs, at 25th and 75th percentiles)

^b^Data was not available on the age at sexual debut for three men, lifetime number of sexual partners of four men, number of sexual acts with study partner in the last month of six men, CD4^+^ T-cell count of one man and viral load measurement for 31 men.

The median age of the men was 36.0 years. All the men were sexually-active, with 54.6 and 37.0% of them being positive for HPV and HIV infections, respectively. Of the men positive for any HPV infection (low-risk (LR) and/or HR-HPV types), 28.5% (37/130) and 71.5% (93/130) were infected with single and multiple HPV types, respectively. HR-HPV infections were detected in 42.9% (102/238) of the men, with 20.6% (21/102) and 79.4% (81/102) infected with single and multiple HR-HPV types, respectively. All the circumcised men in our study were traditionally circumcised.

### Distributions of various taxa in the penile microbiota, their putative oxygen requirements and co-occurrence and co-exclusion patterns

The top 40 most abundant bacterial families identified in the penile microbiota, together with their respective oxygen requirements, are summarised in Additional file 1: Table S1. Bacteria can be categorised according to their oxygen requirements for respiration and growth. Aerobic (*Ae*) bacteria require oxygen for respiration and growth, while anaerobic (*An*) bacteria do not [23]. Facultative anaerobic (*FAn*) bacteria can survive in the presence or absence of oxygen, although growth activity in oxygen-free environment is usually slower [23]. Microaerophilic (*MAe*) bacteria grow in the presence of oxygen but are sensitive to high oxygen concentrations [24]. The most abundant families (at ≥3.5% relative abundance) and their oxygen requirements included *Corynebacteriaceae* (47.19%, *FAn*), *Prevotellaceae* (6.56%, *An*), unclassified *Clostridiales* (5.61%, unidentified), *Porphyromonadaceae* (4.94%, *An*), *Staphylococcaceae* (4.57%, *FAn*), *Bifidobacteriaceae* (3.88%, *An*/*FAn*), and *Lactobacillaceae* (3.81%, microaerophilic (*MAe*)/*FAn*). Of the top 40 bacterial families, *FAn* bacteria were the most dominant (53.39%) followed by *An* bacteria (18.16%) and *Ae* (9.86%). The relative abundances of families with oxygen requirement categorised as *An*/*FAn* and *MAe* were 5.66% and, 3.98%, respectively. Families with unidentified oxygen requirement had an overall relative abundance of 7.33%. The relative abundances of families with *MAe* (0.18%) and *MAe*/*Ae* (0.26%) were very low.

To assess the potential relationships between the bacterial families in our study, we performed correlations between the most abundant bacterial families detected in the penile microbiota, using the Spearman’s rank correlation. Two families, *Pseudomonadaceae* and *Oxalobacteraceae,* that were not abundant in our study, were also included in the analysis as they have been shown to be positively correlated with each other in a previous penile microbiome study [12].

**Fig. 1:**
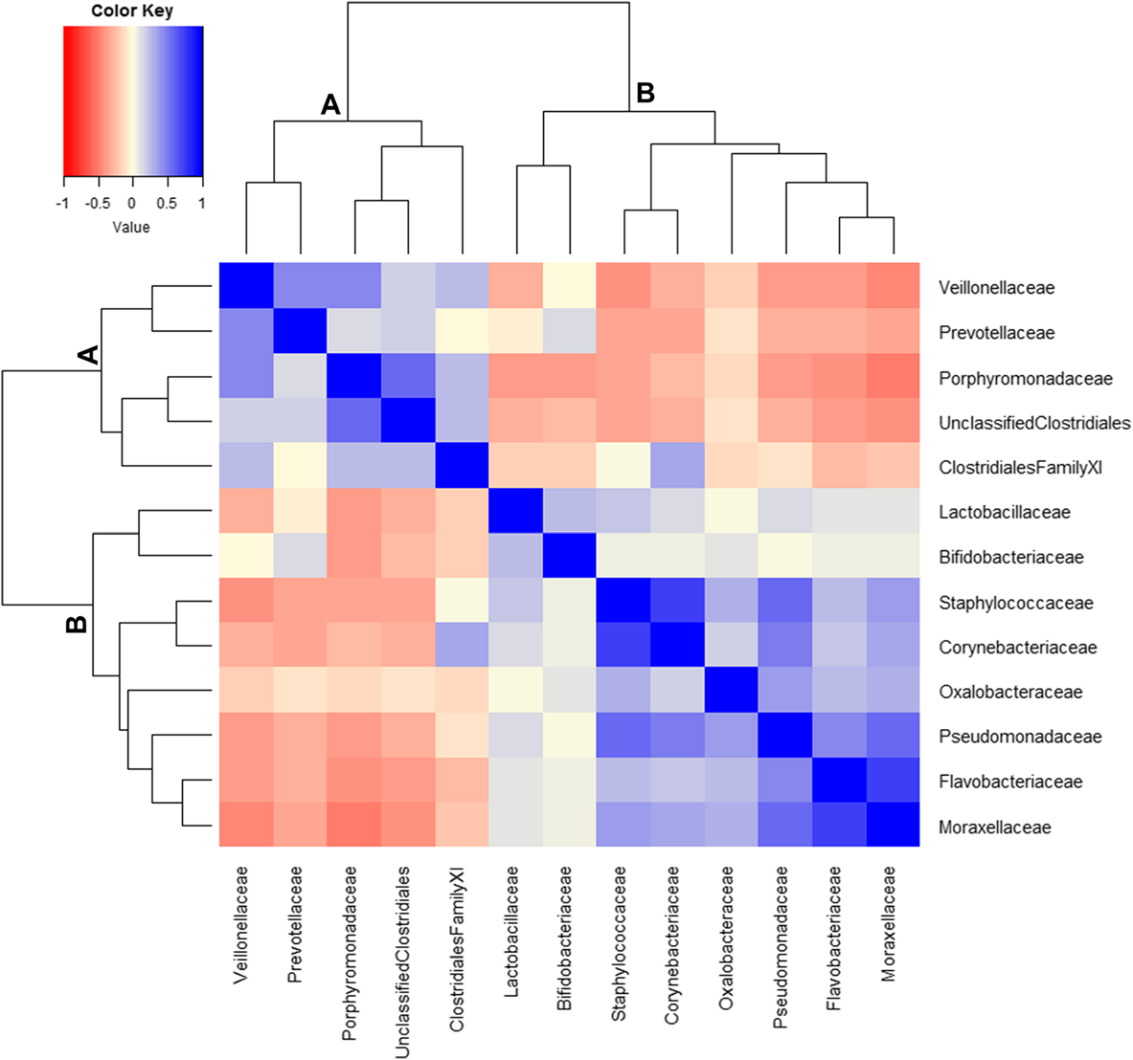
Correlogram of 13 bacterial families showing co-occurrence and co-exclusion patterns. These were computed by Spearman’s rank correlation between the families. The correlation coefficients range from − 1 (red; co-exclusions relationships) to + 1 (blue; co-occurrence relationships), hence, high negative and positive coefficient values indicate strong correlations. The blue diagonal line represents correlations of + 1. White shows absence of bacterial relationships

The correlogram depicting the observed relationships between the bacterial families (positive and negative) is shown in Fig. 1. Two major clusters of bacterial families (designated as A and B) were evident in the correlogram, as indicated by the first bifurcation of the clustering dendrogram (Fig. 1). Cluster-A comprised of *Veillonellaceae*, *Prevotellaceae*, *Porphyromonadaceae*, unclassified *Clostridiales*, and *Clostridiales Incertae Sedis XI*. These bacterial families were positively correlated with one another, an indication of niche sharing and metabolic resource overlap. Generally, these bacterial families were negatively correlated with the eight bacterial families in Cluster-B, which included the most abundant family *Corynebacteriaceae as well as Moraxellaceae*, *Flavobacteriaceae*, *Pseudomonadaceae*, *Oxalobacteraceae*, *Staphylococcaceae*, *Bifidobacteriaceae*, and *Lactobacillaceae*, although family *Clostridiales Incertae Sedis XI* (in Cluster-A) was positively correlated with family *Corynebacteriaceae* (in Cluster-B). Negative interactions between families in Cluster-A and Cluster-B suggest that these bacteria may be competing against one another and may therefore co-exclude one another in the penile niche. The bacterial families in Cluster-B were positively correlated with one another, with *Bifidobacteriaceae* and *Lactobacillaceae* exhibiting the weakest positive interactions (Spearman r < 0.4) with the other families in this cluster. These two bacterial families clustered together following the first bifurcation of Cluster-B. Although *Bifidobacteriaceae* and *Prevotellaceae* were in different clusters (Cluster-B and Cluster-A, respectively), they were positively correlated with one another.

A total of 650 genera were detected. Only 50 of the 650 genera had ≥0.08% relative abundance; they were distributed among 6 phyla. The most abundant genera were *Corynebacterium* (47.12%), *Prevotella* (6.50%), unclassified *Clostridiales* (5.61%), *Porphyromonas* (4.85%), *Staphylococcus* (4.39%), *Lactobacillus* (3.81%), *Gardnerella* (3.78%), *Chryseobacterium* (2.44%), *Acinetobacter* (2.27%), and *Negativicoccus* (1.86%). Additional file 2: Figure S1 shows a relative abundance heatmap of the most abundant genera (≥0.08%) grouped into their respective phyla.

### Establishment of penile microbiota community state types

**Fig. 2:**
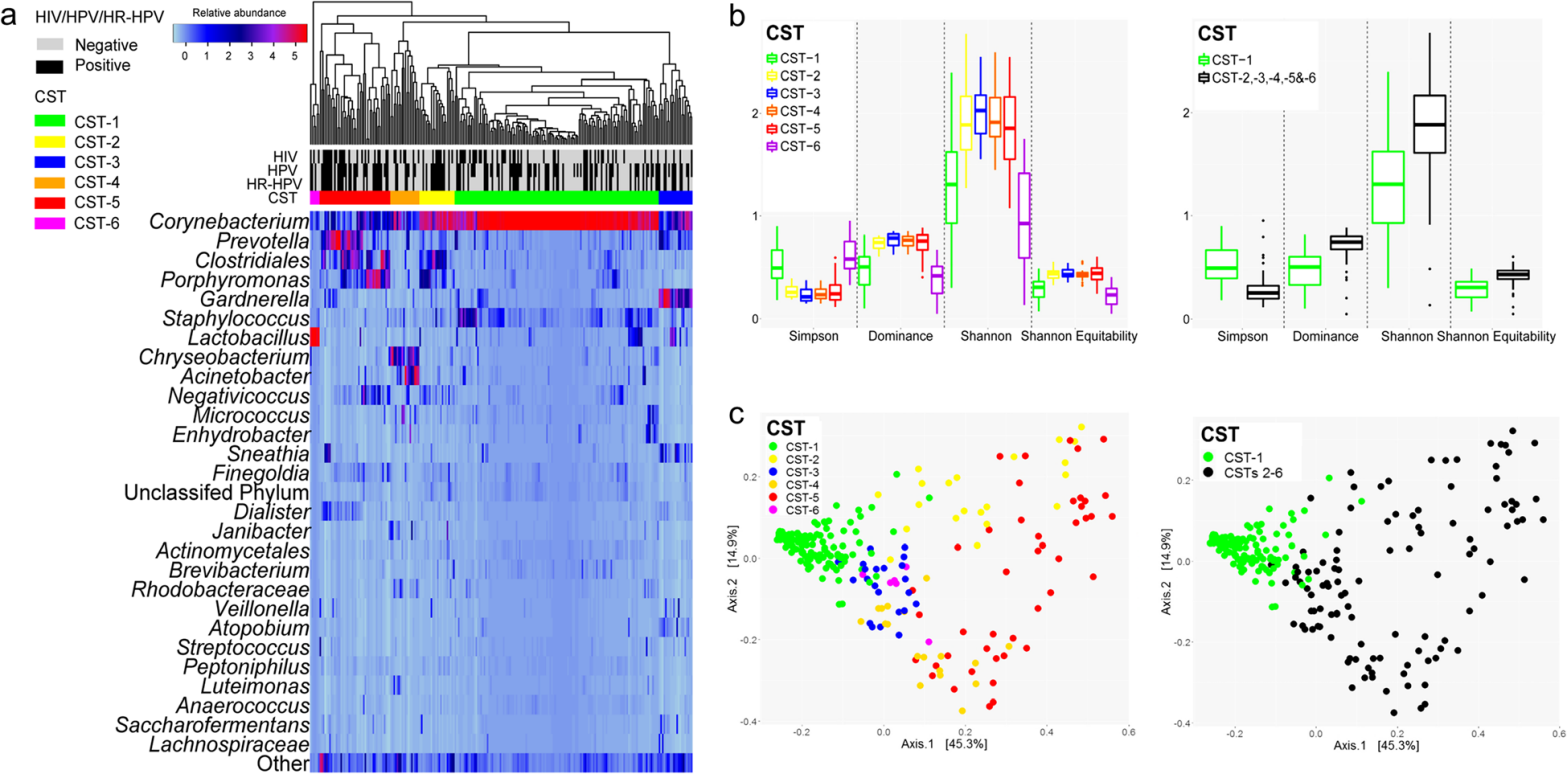
Community state types (CSTs) identified in the penile microbiota. **a**) Heatmap of the relative abundances of bacterial taxa in the 238 penile microbiota. Taxa names of bacteria are shown on the left of the heatmap. The “Other” comprised of pooled bacteria at < 0.31% relative abundance each (*n* = 622, total relative abundance: 6.49%). Rows represent the bacterial taxa and columns the samples. The colour key for the relative abundances is indicated in the upper right corner. The human immunodeficiency virus (HIV), human papillomavirus (HPV) and high-risk human papillomavirus (HR-HPV) infection status of the men are indicated. The dendrogram based on average linkage hierarchical clustering of the Bray-Curtis dissimilarity matrix is shown and was used to define the six community state types (CST-1 through − 6). **b**) Comparison of the alpha diversity of penile microbiota grouped by individual and pooled CSTs. **c**) Comparison of beta diversity (UniFrac distance) of the penile microbiota grouped by individual and pooled CSTs

To define community state types (CSTs), the penile samples were hierarchically clustered based on the microbiota composition. The clustering and heatmap of the relative abundance of the bacterial taxa across the 238 penile samples is shown in Fig. 2a. Based on the Bray-Curtis dissimilarity index, the penile bacterial communities clustered into six CSTs. These CSTs were numbered from 1 to 6.

Table 2 summarises the prevalence of CSTs and the relative abundance of the most abundant bacterial taxa in each CST. *Corynebacterium* was found to the most abundant genera in the penile microbiota (the relative abundance of this genus in each CST is shown in Table 2). The relative abundance of *Corynebacterium* decreased from CST-1 through to CST-6. The most prevalent CST was CST-1; and it was dominated by *Corynebacterium*. CSTs 2–5, which were found in 44.1% (105/238) of the men, were characterised by diverse mixed populations of bacteria and lower relative abundances of *Corynebacterium* than CST-1. The most abundant genera (in decreasing relative abundances) in these CSTs were *Corynebacterium*, unclassified *Clostridiales*, and *Porphyromonas* in CST-2; *Gardnerella* and *Corynebacterium* in CST-3; *Chryseobacterium*, *Corynebacterium*, and *Acinetobacter* in CST-4; and *Prevotella*, unclassified *Clostridiales*, *Corynebacterium*, and *Porphyromonas* in CST-5. The remaining CST, CST-6, was the least prevalent CST and was dominated by *Lactobacillus* with very low relative abundance of *Corynebacterium*. In subsequent analyses, these CSTs were examined individually or in groups based on the dominance of *Corynebacterium* or the diversity of the microbial communities in the CSTs. The specific CST groupings were *Corynebacterium*-dominated (CST-1), non-*Corynebacterium*-dominated (grouped CST-2, − 3, − 4, − 5, and − 6, CSTs 2–6), low diversity (CST-1 and CST-6) and diverse communities (grouped CST-2, − 3, − 4, and − 5, CSTs 2–5).

**Table 2:** Bacterial description of the six established community state types and their prevalences among the 238 heterosexual Black South African men

**Community state type (CST)**	**Dominant bacterial taxa (% relative abundance)**	**Prevalence of CST (% (n/N))**
CST-1	*Corynebacterium* (69.5 (59.5–81.2)) *Staphylococcus* (3.4 (1.5–8.8))	53.4% (127/238)
CST-2	*Corynebacterium* (41.4 (37.9–48.1)) Unclassified *Clostridiales* (12.9 (4.3–22.8)) *Porphyromonas* (8.3 (2.3–23.9))	9.2% (22/238)
CST-3	*Gardnerella* (29.2 (23.3–37.7)) *Corynebacterium* (24.6 (11.2–32.6))	8.8% (21/238)
CST-4	*Chryseobacterium* (22.0 (12.3–34.2)) *Corynebacterium* (16.3 (7.8–29.6)) *Acinetobacter* (15.2 (1.2–36.7))	7.6% (18/238)
CST-5	*Prevotella* (20.1 (2.5–35.7)) Unclassified *Clostridiales* (16.4 (2.1–30.8)) *Corynebacterium* (11.7 (2.8–20.6)) *Porphyromonas* (8.8 (2.7–24.9))	18.5% (44/238)
CST-6	*Lactobacillus* (73.4 (64.1–91.05)) *Corynebacterium* (9.0 (6.4–13.9))	2.5% (6/238)

The relative abundance was expressed as median with interquartile ranges (IQRs, at 25th and 75th percentiles).

The alpha diversity (Fig. 2b) of the highly prevalent *Corynebacterium*-dominated CST-1 was significantly lower than that for CST 2, 3, 4, and 5 (all *p* < 0.0001). Alpha diversity of CTS-6 was significantly lower than CSTs 2, 3, 4, and 5 (all *p* < 0.001). The alpha diversities of CST-1 and CST-6 were not significantly different (*p* = 0.213) and were the least diverse of the six CSTs (Fig. 2). The alpha diversity of *Corynebacterium*-dominated CST-1 was significantly lower than that of non-*Corynebacterium*-dominated CSTs (CSTs 2–6) (*p* < 0.0001) (Fig. 2b). The alpha diversities of these two groups (CST-1 versus CSTs 2–6) were statistically different (*p* < 0.0001): Simpson index: 0.5 (0.4–0.7) versus 0.3 (0.2–0.3), Dominance index: 0.5 (0.3–0.6) versus 0.7 (0.7–0.8), Shannon index: 1.3 (0.9–1.6) versus 1.9 (1.6–2.2), and Shannon Equitability index: 0.3 (0.2–0.4) versus 0.4 (0.4–0.5).

The beta diversities of the penile microbiota were also compared across CSTs using the weighted UniFrac distance metric. PCoA of the beta diversity of the penile microbiota confirmed that the six established CSTs were distinct and clustered according to the unique bacterial communities in each (Fig. 2c; *R*^*2*^ = 0.5569, *p* = 0.001).

Next, the differentially abundant bacterial taxa were determined in *Corynebacterium*-dominated microbiota (CST-1) versus non-*Corynebacterium*-dominated high-diversity microbiota (CSTs 2–5), using linear discriminant analysis (LDA). We found 86 features to be differentially abundant in CST-1 and CSTs 2–5 after the FDR-adjustment (Additional file 3: Table S2). CST-1 and CSTs 2–5 had 40 and 46 enriched features, respectively. These results highlight that the BV-associated bacteria (e.g., *Prevotella*, *Porphyromonas*, *Gardnerella*, *Dialister*, *Finegoldia*, *Mobiluncus*, and *Mycoplasma*) occur in penile microbiota and that these taxa are more predominant in non-*Corynebacterium*-dominated microbiota than in *Corynebacterium*-dominated microbiota.

### Comparison of characteristics of the men with *Corynebacterium*-dominated and non-*Corynebacterium*-dominated penile microbiota

To investigate whether the highly prevalent *Corynebacterium*-dominated penile microbiota (CST-1) were different from non-*Corynebacterium*-dominated penile microbiota (pooled CSTs 2–6), we assessed statistical associations of CSTs with participants’ metadata. The results of comparison of CST-1 versus CSTs 2–6 according to participants’ metadata are shown in Table 3. Among the factors assessed, only HR-HPV infection was significantly different between the two penile microbiota groups (CST-1 and CSTs 2–6). Men with *Corynebacterium*-dominated microbiota had lesser odds of being infected with HR-HPV (odds ratios: HR-HPV: 0.6 [95% CI 0.3–0.9]; *p* = 0.036) than men with non-*Corynebacterium*-dominated microbiota.

**Table 3:** Comparison of the characteristics of men with *Corynebacterium*-dominated and non-*Corynebacterium*-dominated penile microbiota

**Characteristic**	***Corynebacterium*-dominated microbiota (CST-1)** **(*N*** **= 127)**	**Non-*Corynebacterium*-dominated microbiota(CSTs2–6)** **(*N*** **= 111)**	***p-*value**^**#**^
Age (years)	37.0 (31.0–44.0)	36.0 (30.0–44.0)	0.597
Age at sexual debut (years)^a^	17.0 (15.0–18.0)	16.0 (15.0–18.0)	0.975
Number of lifetime sexual partners	6.0 (3.0–12.0)	7.0 (3.0–14.0)	0.693
Number of sexual acts with study partner in last month^a^	4.0 (2.0–10.0)	4.0 (2.3–10.0)	0.763
Current use of condom (% (n/N))	64.7 (77/119)	63.4 (64/101)	0.888
HPV-positive (%(n/N))	51.2 (65/127)	58.6 (65/111)	0.297
HR-HPV-positive (%(n/N))	36.2 (46/127)	50.5 (56/111)	**0.036**
Multiple HPV infection (%(n/N))	64.6 (42/65)	78.5 (51/65)	0.080
Multiple HR-HPV infection (%(n/N))	73.9 (34/46)	80.4 (45/56)	0.438
HIV-positive (% (n/N))	33.9 (43/127)	40.5 (45/111)	0.346
CD4^+^ T-cell count if HIV-positive (cells/μl)^a^	348.5 (240.5–479.8)	308 (217.0–484.5)	0.471
HIV viral load in HIV-positive men (copies/ml)^a^	4.0 log_10_ (3.1–4.7)	4.2 log_10_ (3.8–4.8)	0.376
Circumcised (% (n/N))	93.6 (117/125)	95.1 (98/103)	0.776
Cigarette use (% (n/N))			
Never smoked	15.7(20/127)	18.2 (20/110)	0.879
Ex-smoker	17.3 (22/127)	16.4 (18/110	
Current smoker	66.9 (85/127)	65.5 (72/110)	

Abbreviations: *HPV* Human papillomavirus, *HR-HPV* High-risk human papillomavirus, *HIV* Human immunodeficiency virus, *CST* Community state type

^#^*p*-values are shown for the comparison of HPV-negative and HPV-positive men. Associations of continuous variables (expressed as medians with interquartile ranges (IQRs, at 25th and 75th percentiles)) and categorical variables were computed by Mann-Whitney unpaired and Chi-square/Fisher’s exact tests, respectively. Significant *p*-values (< 0.05) are shown in bold

^a^Data was not available on the age at sexual debut for three men (three with *Corynebacterium*-dominated microbiota), lifetime number of sexual partners of four men (one with *Corynebacterium*-dominated microbiota and three with non-*Corynebacterium*-dominated microbiota), number of sexual acts with study partner in the last month of six men (three with *Corynebacterium*-dominated microbiota and three with non-*Corynebacterium*-dominated microbiota), CD4^+^ T-cell count of one man (with *Corynebacterium*-dominated microbiota) and viral load measurement for thirty one men (seven with *Corynebacterium*-dominated microbiota and twenty four with non-*Corynebacterium*-dominated microbiota).

Since men with *Corynebacterium*-dominated microbiota (CST-1) had less HR-HPV (Table 3) compared to men with non-*Corynebacterium*-dominated microbiota (CSTs 2–6), we next compared the prevalence of HPV, HR-HPV, and HIV infections in CST-1 versus each of the other CSTs (with sufficient number of men, *n* ≥ 20) (Table 4). Men in CST-5 were significantly more likely to be infected with HPV or HR-HPV than men in CST-1 (Table 4, *p* = 0.034 and *p* = 0.005, respectively).

**Table 4:** Association of selected community state types with prevalent HPV, HR-HPV, and HIV infections

**CST**	**HPV prevalence** **(% (n/N))**	**OR (95% CI)** **(*p*-value**^**#**^**)**	**HR-HPV prevalence** **(% (n/N))**	**OR (95% CI)** **(*p*-value**^**#**^**)**	**HIV prevalence** **(% (n/N))**	**OR (95% CI)** **(*p*-value**^**#**^**)**
CST-1	51.2 (65/127)	Ref	36.2 (46/127)	Ref	33.9 (43/127)	Ref
CST-2	54.5 (12/22)	1.1 (0.5–2.8) 0.821	54.5 (12/22)	2.1 (0.8–5.3) 0.154	40.9 (9/22)	1.4 (0.5–3.4) 0.629
CST-3	57.1 (12/21)	1.3 (0.5–3.2) 0.645	42.9 (9/21)	1.3 (0.5–3.4) 0.629	19.0 (4/21)	0.5 (0.1–1.5) 0.213
CST-5	70.5 (31/44)	2.3 (1.1–4.7) **0.034**	61.4 (27/44)	2.8 (1.4–5.7) **0.005**	50.0 (22/44)	2.0 (1.0–3.9) 0.072

Abbreviations: *CST* Community state type, *HPV* Human papillomavirus, *HR-HPV* High-risk human papillomavirus, *HIV* Human immunodeficiency virus, *OD* Odds ratio, *CI* Confidence interval, *Ref* Reference

^#^*p*-values were computed using Fisher’s exact test. Significant *p*-values (< 0.05) are shown in **bold**

No statistical difference in HIV prevalence in the CSTs was observed, although a trend towards higher HIV prevalence in CST-5 compared to CST-1 was observed (Table 4, *p* = 0.072).

### Alpha and beta diversity of the penile microbiota by HIV and HPV status.

**Fig. 3:**
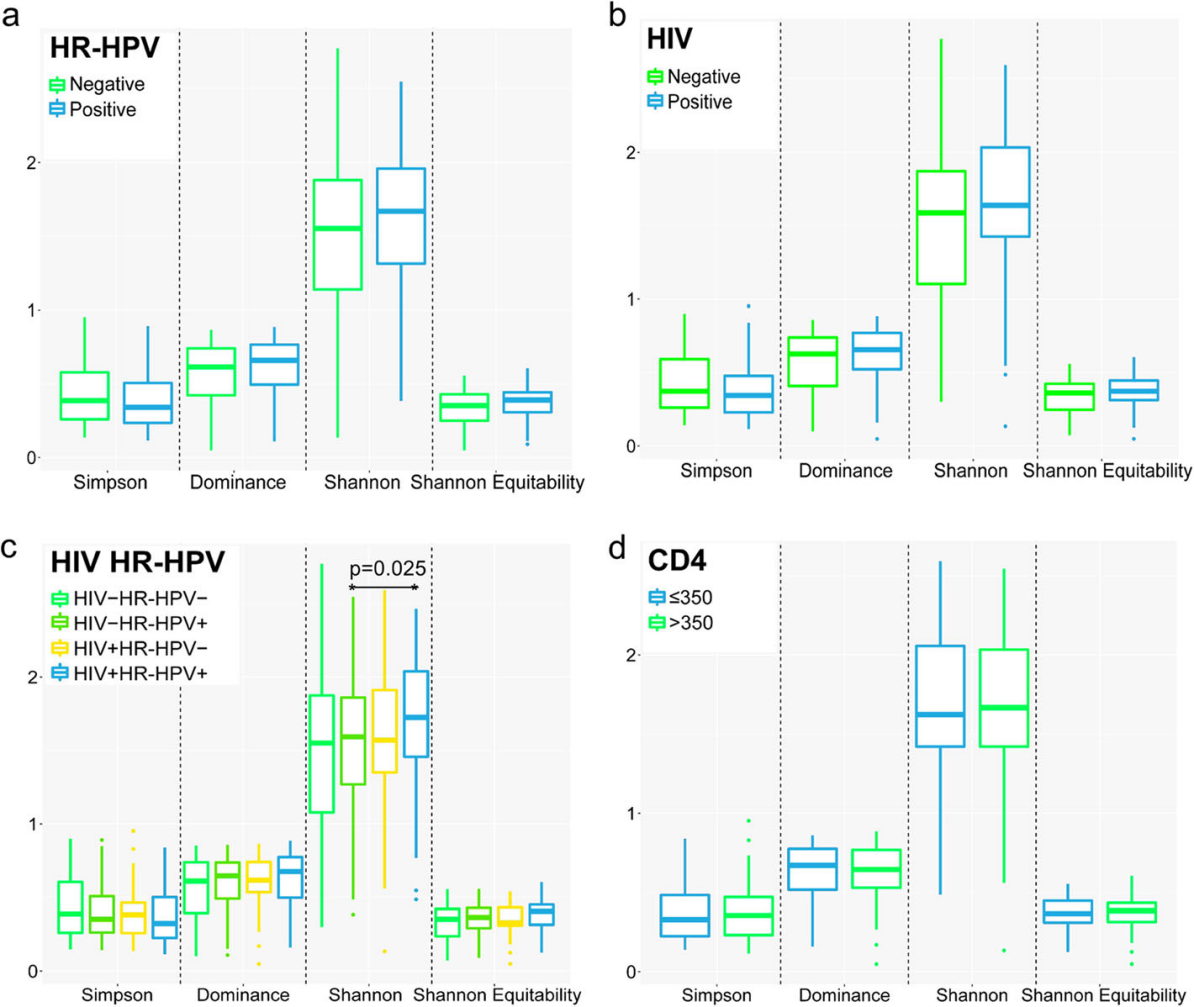
Alpha diversity measures of penile microbiota. Comparison of the alpha diversity of penile microbiota grouped by **a**) high-risk human papillomavirus (HR-HPV) infection status, **b**) human immunodeficiency virus (HIV) infection status, **c**) HR-HPV and HIV infection status, and **d**) CD4^+^ T-cell count status. In each plot, the box ranges from the first to the third quartile, with the median represented by the horizontal line. The whiskers extend to the smallest and largest non-outliers and outliers are represented by dots

To assess the within-sample taxonomic diversities, we calculated the alpha diversity indices of the penile bacterial communities. The alpha diversity across HPV status, HR-HPV status, HIV status and CD4^+^ T-cell count were compared (Fig. 3).

The median alpha diversities of the penile microbiota of men with and without HR-HPV infection were not significantly different (Shannon: 1.6 (1.3–2.0) versus 1.5 (1.1–1.9), respectively (*p* = 0.149)) (Fig. 3a). Similarly, non-significant differences (*p* = 0.296) were obtained for HPV-negative (1.5 (1.1–1.9)) versus HPV-positive (1.6 (1.3–1.9)) groups (Additional file 4: Figure S2a).

The alpha diversities were not significantly different between men with and without HIV infection (Fig. 3b), although there was a trend towards increased microbiota diversity in HIV-positive men compared to HIV-negative men (Shannon: 1.6 (1.4–2.0) versus 1.6 (1.1–1.9) respectively, *p* = 0.050).

Further, we compared the diversity of the penile microbiota of men co-infected with both HIV and HPV (HIV + HPV+), HIV-positive men without HPV infection (HIV + HPV-), HIV-negative men with HPV infection (HIV-HPV+), and men without HIV and HPV co-infections (HIV-HPV-). The penile microbiota of HIV + HPV+ men had statistically higher alpha diversities (Shannon: 1.7 (1.4–2.1) than HIV-HPV- men (Shannon: 1.6 (1.1–1.9); *p* = 0.037) and HIV-HPV+ men (Shannon: 1.6 (1.1–1.9); *p* = 0.045) (Additional file 4: Figure S2b). Alpha diversities of penile microbiota of HIV + HPV- men (Shannon: 1.5 (1.3–1.8) did not significantly vary from HIV + HPV+ men (*p* = 0.182), HIV-HPV- men (*p* = 0.828), and HIV-HPV+ men (*p* = 0.875). Similarly, alpha diversities did not differ between HIV-HPV+ men and HIV-HPV- men (*p* = 0.918). However, HIV-positive men with HR-HPV infections had significantly higher alpha diversity than HIV-negative men with HR-HPV (Fig. 3c, Shannon: 1.7 (1.5–2.1) versus 1.6 (1.1–1.9), respectively; *p* = 0.025).

The alpha diversities of the HIV-positive men with ≤350 CD4^+^ T-cell count and those with > 350 CD4^+^ T-cell count were not significantly different (Shannon: 1.6 (1.4–2.1) versus 1.7 (1.4–2.0), respectively; *p* = 0.873) (Fig. 3d).

**Fig. 4:**
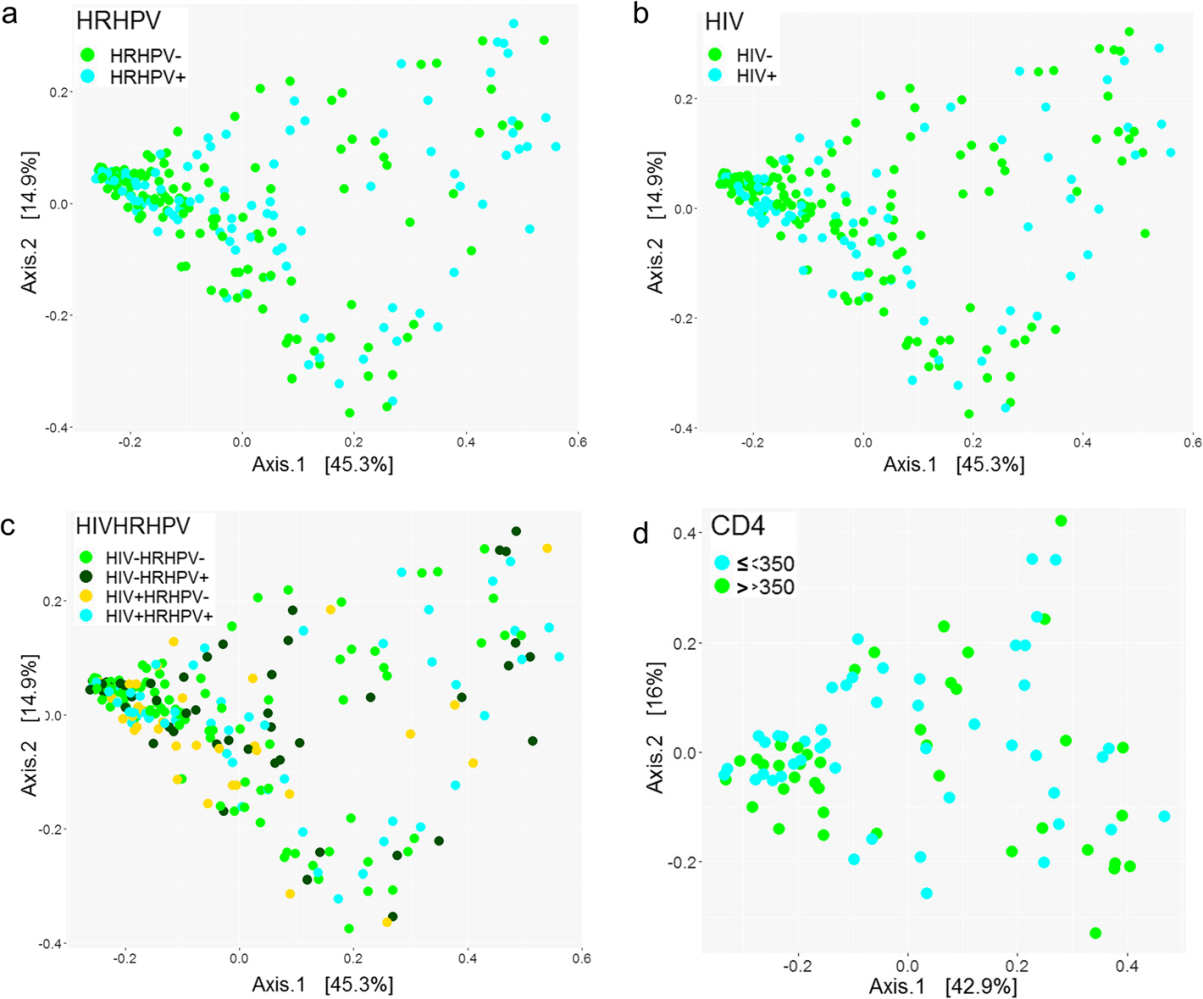
Beta diversity of the penile microbiota. Principal Coordinates Analysis (PCoA) plots of the weighted UniFrac distances of the penile microbiota coloured according to **a**) high-risk human papillomavirus (HR-HPV) infection status, **b**) human immunodeficiency virus (HIV) infection status, **c**) HIV and HR-HPV infection, and **d**) CD4^+^ T-cell count status. The first two principal coordinate axes of variations and the percentage variation explained by each (Axis.1: 45.3% and Axis.2: 14.9% (a-c); and Axis.1: 42.9% and Axis.2: 16.0% (**d**)) are shown. Each solid point is a bacterial community

Beta diversities of the penile microbiota were computed using UniFrac distances and compared among HPV, HR-HPV, HIV, and CD4^+^ T-cell count groups. The resultant 2D PCoA plots of clustering based on weighted UniFrac distances are shown in Fig. 4.

We observed that there was no separation of the samples when stratified according to HPV status (Additional file 4: Figure S2c; *R*^*2*^ = 0.0088, *p* = 0.069). The beta diversity of the communities in HR-HPV-uninfected men was significantly different from that of HR-HPV-infected men (Fig. 4a; *R*^*2*^ = 0.0123, *p* = 0.011). About 63.8% (81/127) of the men without HR-HPV infections had *Corynebacterium*-dominated penile microbiota that clustered together (Fig. 2c).

The penile bacterial communities of men with and without HIV infection showed no distinct separation, indicating that HIV did not impact the clustering of these communities (Fig. 4b; *R*^*2*^ = 0.0061, *p* = 0.189).

Similarly, co-infection with HIV and HPV did not affect the clustering of the bacterial communities (Additional file 4: Figure S2d; *R*^*2*^ = 0.0192, *p* = 0.077). However, the samples clustered according to HIV and HR-HPV co-infection status (Fig. 4c; *R*^*2*^ = 0.0231, *p* = 0.049).

No difference in beta diversities was observed in communities from HIV-positive men with ≤350 CD4^+^ T-cell count and those with > 350 CD4^+^ T-cell count (Fig. 4d; *R*^*2*^ = 0.0077, *p* = 0.129), thus suggesting no relationship of CD4^+^ T-cell count with community structure.

### Differential bacterial relative abundance and potential biomarkers for HR-HPV and HIV infections

Bacterial taxa that were differentially abundant in the penile microbiota of men with and without HR-HPV and HIV infections were analysed using Linear Discriminant Analysis (LDA) effect size (LEfSe) algorithm [25].

**Fig. 5:**
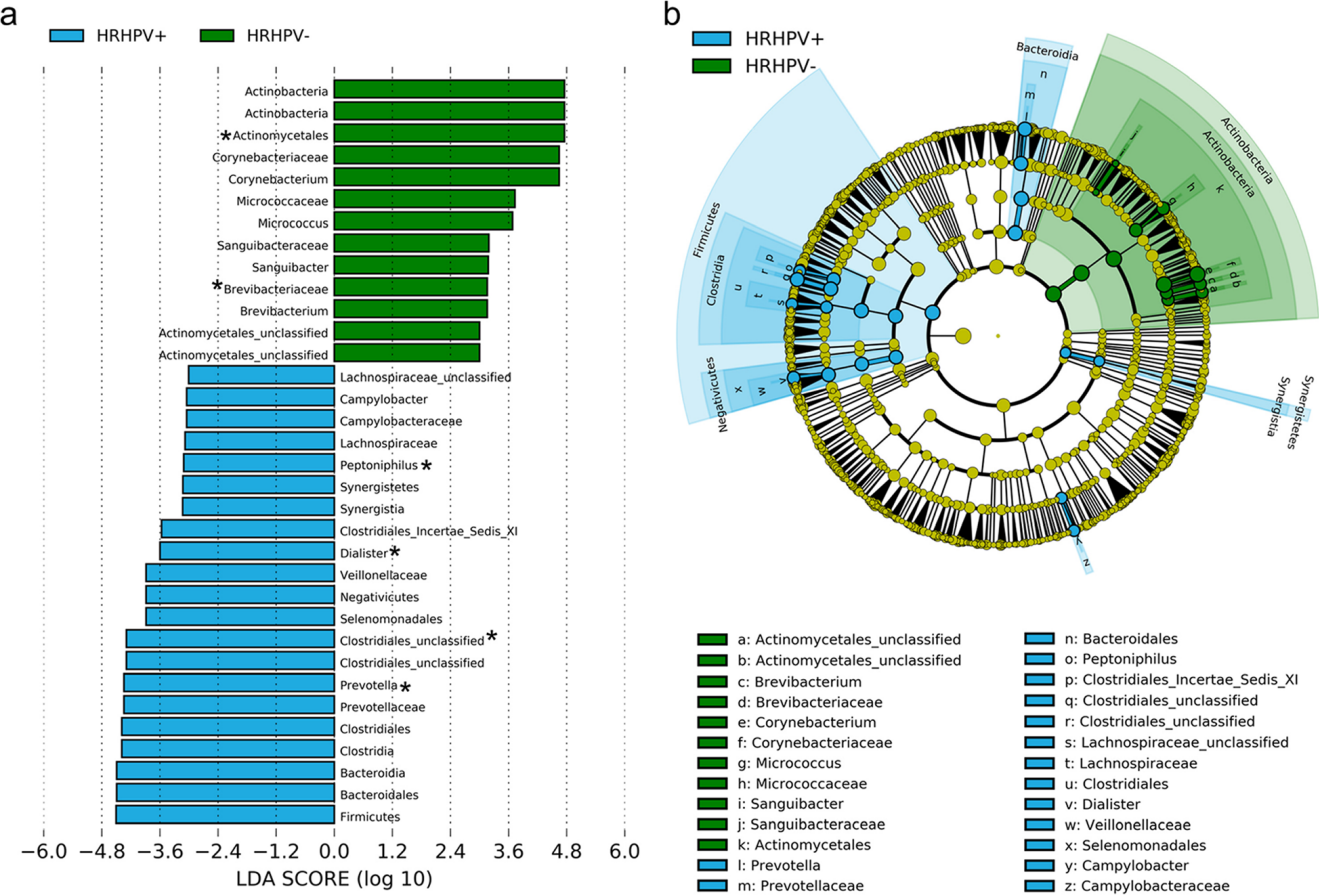
Potential biomarkers for HR-HPV infection by LEfSe. **a**) Histogram of differentially abundant taxa in penile microbiota of men with and without HR-HPV infections, and **b**) a six-level cladogram with a taxonomic hierarchical structure indicating differentially abundant taxa in penile microbiota of men with and without HR-HPV infections. Each coloured solid represents a taxon and its diameter is proportional to the taxon’s relative abundance. Blue and green colours represent statistically significant taxon ranks in HR-HPV-positive and negative group, respectively. For visualisation purposes, only differentially abundant features at logarithmic LDA scores > 3.0 or < − 3.0 are shown. Asterisks in the histogram indicate significantly differentially abundant taxa with q < 0.2 after FDR correction

Biomarkers for HR-HPV were first assessed irrespective of HIV status. A total of 78 bacterial features for HR-HPV were detected at LDA > 2.0 or < − 2.0. The most significant of these biomarkers for HR-HPV infection (LDA > 3.0, *p* < 0.05) are shown in Fig. 5, with a histogram showing the identified biomarkers ranked by effect size (Fig. 5a) and a six-level cladogram representing the taxonomic hierarchical structure of the identified biomarkers (Fig. 5b). In HR-HPV-infected men, families *Campylobacteraceae*, *Prevotellaceae*, and *Veillonellaceae* were found to be in greater relative abundances than in men without infection. The relative abundances of *Prevotella*, *Peptoniphilus*, and *Dialister* were found to be significantly greater in the penile microbiota of men with HR-HPV infection than uninfected men, after correction for multiple comparisons (q < 0.2). In uninfected men, the classified genera that were detected at greater relative abundances (LDA < -3.0, *p* < 0.05) included *Corynebacterium*, *Micrococcus*, *Sanguibacter*, and *Brevibacterium*. After adjustment for multiple comparisons, the relative abundances of the family *Brevibacteriaceae* and order *Actinomycetales* were significantly higher in uninfected men compared to HR-HPV-infected men.

Differentially abundant taxa in the penile microbiota of HR-HPV men were additionally assessed in HIV-negative men only. When only HIV-negative men were considered, the genera that had greater relative abundances in men with HR-HPV infection than in men without HR-HPV infection (LDA > 2.0, *p* < 0.05) included *Jonquetella*, unclassified *Clostridiales*, unclassified *Campylobacteraceae*, *Paenalcaligenes*, *Pimelobacter*, unclassified *Porphyromonadaceae*, and *Blastomonas* (Additional file 5: Figure S3). These differences in relative abundances were found not to be significant after adjustment for multiple testing. The order *Actinomycetales*, as well as the genera *Brevibacterium* and *Brachybacterium* within this order, were however found to be significantly enriched (q < 0.2) in uninfected men compared to HR-HPV-infected HIV-negative men after adjustment for multiple comparisons (Additional file 5: Figure S3).

**Fig. 6:**
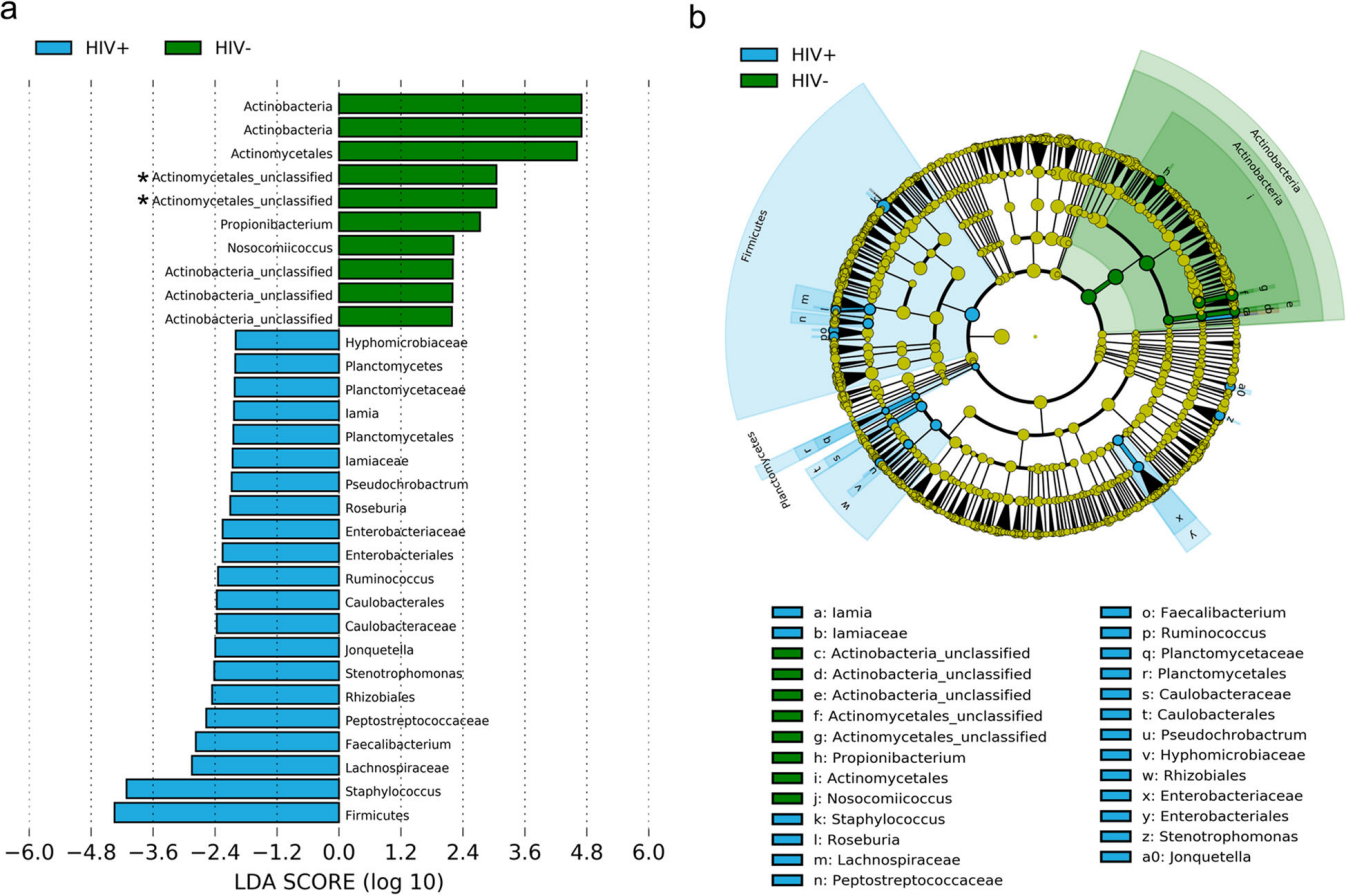
Potential biomarkers for HIV infection by LEfSe. **a**) Histogram of differentially abundant taxa in penile microbiota of men with and without human immunodeficiency virus (HIV) infection, and **b**) a six-level cladogram with a taxonomic hierarchical structure. Each coloured solid represents a taxon and its diameter is proportional to the taxon’s relative abundance. Blue and green solids represent statistically significant taxon ranks in men with and without HIV infection, respectively. Only differentially abundant features at logarithmic LDA scores > 2.0 or < − 2.0 are shown. Asterisks indicate significantly differentially abundant taxa with q < 0.2 after FDR correction

Bacterial taxa that were differentially abundant in the penile microbiota of HIV-positive and HIV-negative men were also evaluated using LEfSe (Fig. 6). The analysis identified 31 differentially abundant (LDA scores > 2.0 or < − 2.0, *p* < 0.05) bacterial features in the penile microbiota, which are presented in a histogram according to effect size (Fig. 6a) and a cladogram with a taxonomic hierarchical structure (Fig. 6b). Twenty one of these features were found in men who were infected with HIV while the rest of the taxa (*n* = 10) were found in men who were HIV-negative. Men with HIV infections had greater relative abundances of the genera *Staphylococcus*, *Faecalibacterium*, *Strenotrophominas*, *Jonquetella*, *Ruminococcus*, *Roseburia*, *Pseudochrobactrum*, and *Lamia* than men without HIV infections; although after correction for multiple testing the differences were not significant. On the other hand, the relative abundances of *Propionibacterium*, *Nosocomiicoccus*, and unclassified genera in the class Actinobacteria and order *Actinomycetales*, were greater in HIV-negative men than HIV-positive men. None of the predominant bacterial families in the penile microbiota (*Corynebacteriaceae*, *Prevotellaceae*, and unclassified *Clostridiales*) were found to differ significantly in relative abundances between HIV-infected and uninfected men. The relative abundances of the predominant penile bacterial families stratified by HIV infection status are shown in Additional file 1: Table S1. *Corynebacteriaceae*, *Prevotellaceae*, and unclassified *Clostridiales* were found to be the most common families regardless of HIV status. Each of these families occurred at relative abundances of between 5.2 and 49.7%.

## Discussion

In this study, we examined the penile microbiota of 238 Black South African men. To date, this is the largest cross-sectional study using a culture-independent approach to detail the bacterial communities of the penile skin. Sampling of the penile skin microbiota was done by swabbing of the glans, coronal sulci (if present), and shaft of the penis. To date only one other study, by Zozaya and co-workers (2016) [26], has used a similar sampling method. The other studies, swabbed either coronal sulcus alone [5, 12, 13, 27, 28], glans alone [29], or both coronal sulcus and glans [30]. Thus, our sampling method together with Zozaya and co-workers’ (2016) [26], provides a more comprehensive picture of the microbiota of the whole penis.

The most common bacterial families in the penile microbiota were, in order of decreasing relative abundances, *Corynebacteriaceae*, *Prevotellaceae*, unclassified *Clostridiales*, *Porphyromonadaceae*, and *Staphylococcaceae*. Facultative anaerobic (*FAn*) families (*n* = 7) were the most abundant families with a mean relative abundance over 50% of the penile microbiota, with the family *Corynebacteriaceae* contributing 47%. The next most abundant group was anaerobic (*An)* families (18.2%). High predominance of *FAn* and lower predominance of *An* families have previously been significantly associated with post-circumcision status [12]. Among the most common families, strong positive correlations were observed between *Corynebacteriaceae* and *Staphylococcaceae*, both facultative anaerobes. The positive correlations highlight a possibility of cooperative interaction through metabolic resource overlap [31]. These two families in turn had strong negative correlations with the anaerobic *Prevotellaceae* and *Porphyromonadaceae*, as well as the unclassified *Clostridiales*. Negative correlations indicate the presence of competition for resources [31, 32] and subniche differentiation [32]. This suggests that *Corynebacteriaceae* and *Staphylococcaceae* might be the main competitors against the other three predominant families.

We observed that *Corynebacterium* was the most prevalent (100%) and abundant (47.1%) genus. The relative abundance of *Corynebacterium* in our study is similar to that (45.9%) observed on the penile skin by Zozaya and colleagues (2016) [26]. Further, Zozaya and colleagues (2016) [26] reported that the relative abundance of *Corynebacterium* was lower in uncircumcised men (31.7%) compared to circumcised men (55.2%). Thus, the relative abundance of *Corynebacterium* in our cohort of mostly traditionally circumcised men was intermediate to these published results and higher than uncircumcised men [26]. Greater abundances of *Corynebacterium* have also been reported in coronal sulci microbiota post-circumcision and in circumcised men [12, 13]. For example, medical male circumcision was found to increase the proportional abundances of *Corynebacterium* in the coronal sulci microbiota of Ugandan men [12]. Apart from colonising the coronal sulci, *Corynebacterium* can also colonise the semen microbiota of men in appreciable abundances [33, 34].

A cluster analysis of the penile microbiota revealed that the penile bacterial communities could be represented by six distinct community state types (CSTs) with different bacterial diversities. Over 50% of the men had *Corynebacterium*-dominated penile microbiota (CST-1). Six of the 238 men (2.5%) had penile microbiota dominated by *Lactobacillus* (CST-6). Although *Lactobacillus* has been reported to inhabit the human penis [27], its origin, especially among sexually-active men, could be the vagina. This is because of the predominance of *Lactobacillus* in the vaginas of reproductive-age women [35] and the fact that *Lactobacillus* has been reported to considerably increase in abundance in the penile microbiota following unprotected sexual exposure [29]. The remaining 44.1% of the men (CSTs 2–5) had diverse communities not dominated by *Corynebacterium* or *Lactobacillus*. The diverse CSTs, CSTs 2–5, had significantly lower relative abundances of *Corynebacterium* and *Staphylococcus* and greater relative abundances of several taxa, particularly the BV-associated bacteria compared to CST-1 (Additional file 3: Table S2). Comparisons of the alpha diversities of the communities in the CSTs indicated that CST-1 and CST-6 were significantly less diverse than CSTs 2–5.

The distinct CSTs observed in the current study may relate to differences in penile moisture, sebum levels and oxygen availability. The penis provides several microenvironments that differ in levels of moisture, sebum and oxygen that may support distinct microbiota [5, 12, 14, 26, 27, 36]. The coronal sulci and distal urethra, for example, were found in young adolescent men to harbor distinct bacterial communities [36]. *Corynebacterium* and *Staphylococcus* were abundant members of the coronal sulci microbiota, *Streptococcus* and *Lactobacillus* were the most abundant in the distal urethra of adolescent males [36]. The coronal sulci microbiota was further found to be more stable over time compared to the microbiota of the distal urethra [36]. *Corynebacterium* and *Staphylococcus*, the predominant bacteria in CST-1, are abundant members of the superficial skin microbiota associated with moist sites [37]. Several recent studies have also revealed that many bacterial genera in the penile microbiota are associated with the vaginal flora of both healthy women and women with BV, with many speculating that sexual exchange of urogenital bacteria is likely broader than previously thought [38, 39]. Our observation that various BV-associated genera and *Lactobacillus* are represented in CSTs 3–6 may therefore reflect sexual exchange of these taxa. Testing of this in future studies will require longitudinal sampling of the penile skin microbiota and surveillance of the microbiota in relation to incident sexual exposure.

Men with *Corynebacterium*-dominated penile skin microbiota (CST-1) had less HR-HPV infections than those in CSTs 2–6. Among the diverse CSTs, CST-5 was characterised by a low relative abundance of *Corynebacterium* and greater relative abundances of *Prevotella*, unclassified *Clostridiales* and *Porphyromonas*; CST-5 was associated with greater numbers of HPV and HR-HPV infections when compared to CST-1. While no previous studies have been carried out to examine associations of penile microbiota with HPV, *Corynebacterium* is known to be abundant in post-circumcised penile swabs [13, 26]; and it was shown that circumcision is associated with protection against penile HPV infection [8, 9, 11, 40–42]. One trial of male circumcision for HIV/STI prevention associated circumcision with reduced prevalent HPV in penile shaft and coronal sulci [41]. A randomised trial associated male circumcision with low incidence of multiple HR-HPV and rapid clearance of HR-HPV infections [9]. The biological mechanism for this is unknown. The association between the diverse CSTs and HR-HPV could be due to either an increased susceptibility to HR-HPV genotypes or a delayed clearance of these genotypes. In women, diverse BV-like communities have been described to have the lowest HPV clearance rates [43].

We used one of the machine learning approaches, LEfSe analysis, to identify differentially abundant bacteria in men according to HPV and HIV statuses. We found a significantly greater relative abundances of *Prevotella*, *Peptoniphilus, Dialister*, and unclassified *Clostridiales* (LDA > 2.0, *p* < 0.05, q < 0.2) in the penile microbiota of men with HR-HPV infections compared to uninfected men. To our knowledge, no studies have investigated the associations between penile bacterial taxa and HPV infection. Of note, the BV-associated taxa *Sneathia*, *Prevotella*, *Dialister*, and *Peptoniphilus* have been identified in several vaginal microbiota studies in women [44–47] as biomarkers for either HPV or HR-HPV.

We have observed no significant association between HIV status and penile microbiota CST, alpha diversity, or beta diversity. However, we found that men without HIV infection had significantly lower relative abundance of *Actinomycetales* compared to HIV-infected men. A greater number of men in CST-5 were HIV-infected when compared to CST-1, although the difference was not significant. However, HIV and HPV co-infected men had significantly more diverse penile microbiota than HIV-uninfected men with or without HPV infection. There are no published studies that have investigated the penile microbiome of HIV-infected men. A recent prospective study by Liu and colleagues (2017) [5] on penile microbiota of 182 uncircumcised Ugandan men suggested that penile anaerobic dysbiosis might be a risk factor for acquisition of HIV. The authors demonstrated that the men who seroconverted (25.3%) had significantly greater absolute abundances of anaerobic *Prevotella*, *Dialister*, *Peptoniphilus*, and *Finegoldia* at baseline than men who remained HIV-negative [5]. Moreover, these bacteria were found to trigger proinflammatory responses that are thought to facilitate infection by HIV [5]; hence, further supporting the strong connection between certain genital anaerobic bacteria and inflammatory cytokines [48, 49]. In our study, CST-5 had lower relative abundances of *Corynebacterium* (mean: 12.9%) and was dominated by several BV-associated anaerobic bacteria and/or proinflammatory bacteria such as *Prevotella* (23.0%), unclassified *Clostridiales* (18.5%), and *Porphyromonas* (16.3%). In addition, CST-5 had other bacteria in appreciable relative abundances: *Negativicoccus* (4.8%), *Dialister* (2.6%), *Finegoldia* (2.2%), *Sneathia* (1.9%), *Gardnerella* (1.5%), and *Fusobacterium* (1.1%). BV-associated bacteria were found to be more prevalent and abundant in diverse CSTs; for example, CST-5 was less diverse than CST-1 (CST with *Corynebacterium* dominance) and CST-6 (CST with *Lactobacillus* dominance). Similar observation has also been reported in a penile microbiota study on a Ugandan cohort [27]. Penile anaerobic dysbiosis and/or the predominance of proinflammatory associated bacteria in CST-5 may potentially contribute to the greater prevalence of multiple HPV, HR-HPV, and HIV infections in men in CST-5.

An important consideration in studies examining the penile microbiota of sexually-active heterosexual men is the potential influence of regular exposure of the penile bacterial communities to the vaginal, oral and rectal microbiotas of their sexual partners. A recent case report, for example, found an increase in *Lactobacillus* abundance in the penile microbiota following vaginal sex [29]. Further, a significant proportion of HPV DNA detections may represent depositions from recent unprotected sex with an infected partner [50]. Differences in the time since the last sex act and nature of the last sexual exposure could therefore have impacted the findings reported in the present study. A major limitation of the current study design is that it did not allow for the discrimination between colonising members of the penile microbiota and temporary carriage of organisms following recent sexual exposure. Future studies could address this by including longitudinal sampling in men who abstain from interval sexual exposures. This would allow for analysis of the temporal variation in penile bacterial community membership and structure in the absence of sexual exposure as well as providing an indication of the stability of the reported associations of organisms with HPV infection over time. A further caveat was the use of dry swabs in this study. The sampling of the cutaneous microbiome may have been improved through the more commonly used premoistened swabbing method [37].

## Conclusion

This is the first study to characterise the penile microbiota of traditionally circumcised men and to examine the associations of these microbiota with HPV infection. We found that family *Corynebacteriaceae* and genus *Corynebacterium* were the most abundant bacterial taxa in the penile microbiota of Black South African men. Six CSTs were established, with over 50% of the men having *Corynebacterium*-dominated penile microbiota (CST-1).

Penile microbiota that were not dominated by *Corynebacterium*, and were mostly dominated by BV-associated bacteria (CSTs 2–6), were associated with prevalent HR-HPV infections in Black South African men. Specific penile bacteria (*Prevotella*, *Dialister*, *Peptoniphilus*, and unclassified *Clostridiales*) were identified as significantly enriched in the penile microbiota of HR-HPV-positive men compared to uninfected men. The role, if any, of these bacteria in HR-HPV prevalence, acquisition and clearance should be investigated.

## Methods

### Study design and samples

The present study included 282 randomly selected baseline specimens and their abstracted information from the parent study. Inclusion criteria were samples with baseline information on HIV status, positive human β-globin PCR results (a measure of sample integrity) and sufficient archived sample material for microbiota analyses. Dry Digene swabs (Qiagen, Gaithersburg, Inc., USA) were used to swab the shaft, foreskin (if uncircumcised) and glans of the penis. These were placed in Digene Specimen Transport Medium (STM, Qiagen, Gaithersburg, Inc., USA) and frozen at − 80 °C until DNA extraction. Nucleic acids were isolated from the penile samples using the MagNA Pure Compact Nucleic Acid Isolation kit and the MagNA Pure Compact System (Roche Molecular Diagnostics, Mannheim, Germany). The Roche Linear Array HPV genotyping test (Roche Molecular Diagnostics, Mannheim, Germany) was used to detect 37 HPV types (13 HR and 24 LR types).

### Illumina MiSeq library preparation and sequencing

The hypervariable region V3 and V4 of the 16S rRNA gene were amplified using the universal bacterial primers 319F (5′-CCTACGGGNGGCWGCAG-3′) and 806R (5′-GACTACHVGGGTATCTAATCC-3′) [51]. The 16S rRNA gene libraries for sequencing were prepared according to the 16S rRNA metagenomics protocol for MiSeq System (Illumina, San Diego, CA, USA) [52], with minor modifications [47, 53]. Triplicate PCRs were performed per sample using TaKaRa Ex Taq® DNA Polymerase Hot Start Version (Takara Bio Inc., Japan). Each reaction contained 0.025 U Ex Taq polymerase, Ex Taq buffer (Takara Bio Inc., Japan), 0.8 mM dNTP mixture, 0.56 mg/ml BSA, 400 nM each primer and 100 ng penile DNA. Initial denaturation at 98 °C for 2 min, was followed by 30 cycles of denaturation (98 °C for 20 s), annealing (50 °C for 30 s) and extension (72 °C for 45 s), and a final extension at 72 °C for 10 min. The 282 penile samples were concurrently run with internal controls including negative controls (two nuclease free water and one Digene STM MOCK extraction controls), and positive controls (MOCK communities: two HM-782D (even, low concentration) and one HM-783D (staggered, low concentration) (BEI Resources, Manassas, VA, USA). Triplicate amplicons were pooled and confirmed by 1.5% agarose gel electrophoresis. Pooled amplicons were purified using the Agencourt AMPure XP system (Beckman Coulter, Germany) and quantified using the Quant-iT PicoGreen dsDNA assay (Thermo Fisher Scientific, USA), according to the manufacturer’s instructions. The KAPA HiFi HotStart ReadyMix PCR Kit (KAPA Biosystems, Wilmington, MA, USA) was used to perform the Illumina index PCR. Libraries were pooled in equimolar concentrations and the final library quantified on a Bioanalyzer High Sensitivity Chip (Agilent Technologies, Santa Clara, CA, USA). The libraries from the 282 penile samples and six internal controls were sequenced in three runs on the Illumina MiSeq using a paired-end 300-bp protocol and v3 reagents at the Center for Genomic Regulation (CRG, Barcelona, Spain).

### 16S rRNA gene sequence dataset analyses

The qualities of the raw sequenced reads were assessed by FastQC v0.11.2 [54]. The sequenced reads were processed using mothur v1.37.6 [55] using the standard operating procedure (SOP) guidelines [56], with slight modifications. The mothur pipeline had been validated as previously described [57]. The forward and reverse reads were assembled into contiguous reads. The sequences that had length below 439 bp, above 466 bp, and more than 4 ambiguous base calls were filtered out. Unique sequences in the fasta-formatted file were selected and then aligned against a reference alignment, SILVA v119 (www.arb-silva.de/), using the Needleman algorithm with kmer of 8 bp (nucleotide substring), − 2 gap opening and − 1 gap extension penalties. The kmer searching was used because it is faster and more reliable than blast and suffix tree template searching methods. Sequences with homopolymeric run longer than 12 bases were removed. The alignments were filtered to remove gaps as they do not have any genetic information. The sequences were further pre-clustered by relative abundance using a pseudo-single linkage algorithm to remove erroneous sequences with > 4 nucleotide mismatches. For additional filtering, singletons were also discarded. Potential chimeras were removed by a de novo method using the UCHIME algorithm [58].

Non-chimeric sequences were assigned taxonomy using the Wang approach of 8 kmers implemented by RDP Classifier [59]. The “trainset9_032012.pds.fasta” and “trainset9_032012.pds.tax” were used as the RDP database sequence and taxonomy files, respectively. The cut-off for bootstrap confidence score for taxonomic assignment was 80%. Lineages of chloroplastic, mitochondrial, archaeal, eukaryotic, and other non-bacterial sequences were removed. This was followed by assessment of the sequencing errors using the MOCK communities [60] in order to determine the reliability of the sequencing procedure (mothur SOP). First, all bacterial sequences were extracted from the MOCK communities using the *get.groups* command. These sequences were then searched for errors against reference sequences of MOCK communities (“HMP_MOCK.v35.fasta”) using the *seq.error* command. Error rate was then calculated using the formula and expressed as a percentage:

$$ Error\ rate=\frac{Sum\  of\ mismatches\ to\ reference}{Sum\  of\ bases\ in\ query} $$

OTUs were defined at a 0.03 cut-off phylogenetic distance using the opticlust clustering algorithm. The quality (completeness) quality of sampling the bacterial communities was measured using Good’s coverage estimate [61]. The coverage calculator in mothur showed that at the recommended 92% Good’s coverage, the majority of the OTUs (potential species), including the low-abundant ones, could be sampled. This coverage was equivalent to a subsampling depth of 13,014 reads (per sample) that showed that could reliably capture the microbiota diversity. The OTU table was therefore normalised by rarefying at 13,014 reads counts per sample. Samples with less than 13,014 reads were not considered further for this analysis. Penile swab samples from 288 South African men were initially selected for microbiota analysis. Two hundred and thirty eight men (84.4%) were finally included in the study. Participants excluded (15.6%, 44/282) were those whose samples had less than 13,014 reads for 16S rRNA gene sequence analyses. The metadata and unrarefied OTU table (with genus taxonomic classifications) are provided in Additional files 6 and 7, respectively.

### Analysis of penile microbiota composition and diversity

The prevalence and relative abundances of the bacteria in the penile microbiota calculated in mothur was summarised at each taxonomic level using customised Python script (*taxonomy_mothur_abundance_silvaDB_v1.2.py*). This was done at the phylum, family, and genus taxonomic ranks.

Alpha and beta diversity analyses of the various metadata categories (HPV, HR-HPV, HIV, CD4, CST, and partner’s BV) were performed using a customised scripts (*biodiversity_calculator.R* and *betaDiv.R*) in RStudio v1.1.447 [62]. Beta diversity was computed using Vegan R package v2.4.3 [63] and phyloseq v1.20.0 [64]. The alpha diversity was computed using Simpson, Dominance, Shannon, and Shannon Equitability indices, while beta diversity was computed using the UniFrac distance matrix were represented using 2D PCoA plots.

The penile microbial communities were clustered into CSTs using the average neighbour linkage method based on Bray-Curtis dissimilarity index calculated using the Vegan R package [63].

### Putative aerotolerance profile of the most abundant penile microbiota families

The aerotolerance (oxygen requirements) of the 40 most abundant families in the penile microbiota were accessed from published penile microbiome literature [12, 13] and extensive literature searches on the PubMed database (https://www.ncbi.nlm.nih.gov/pubmed/). The overall oxygen requirement of each family was then assigned from the oxygen profiles of the genera in that family. For families with genera with different oxygen requirements were considered to have mixed aerotolerance profiles, e.g., *Mae*/*Fan*. For biochemically uncharacterised or taxonomically unclassified families the oxygen requirement was designated as “unidentified”. Finally, to examine the most to least common aerotolerance profile of the 40 most abundant families, we grouped all families with similar oxygen requirements together. For example, *Flavobacteriaceae* and *Moraxellaceae* were grouped together as “aerobic” while *Veillonellaceae* and *Clostridiales Incertae Sedis XI* were grouped as “anaerobic”. We then measured the overall prevalence of aerotolerance profiles of each of these grouped families.

### Co-occurrence and co-exclusion patterns of bacterial families

Positive and negative correlations between the counts of bacterial families in the penile microbiota were assessed using metagenomeSeq v1.12.1 [65, 66]. The families assessed included the eleven most abundant families in the penile microbiota (Additional file 1: Table S1) and two less abundant families (*Pseudomonadaceae* and *Oxalobacteraceae*) that have been previously found to have a positive correlation by Price and colleagues (2010) [12]. A correlogram depicting the correlations between these families was plotted using the *plotCorr* function in the metagenomeSeq v1.12.1 [65, 66].

### Identification of differentially abundant bacterial taxa in HPV- and HIV-infected men

The Linear Discriminant Analysis (LDA) effect size (LEfSe) algorithm v1.0 [25] was used to describe bacterial taxa significantly enriched or depleted in association with positive and negative detection of HIV and/or HPV. The level of statistical significance (*p*-value) was set at 0.05 while the threshold for discriminative features based on the logarithmic LDA score was set at 2.0. False discovery rate (FDR) was used to correct for multiple comparison testing and features with a q-value < 0.2 were considered to be significant. This was computed using MicrobiomeAnalyst [67].

### Statistical analyses

R v3.2.2 (R Core Team 2016) and GraphPad Prism Software v6.01 were used to perform all statistical comparisons. Comparison of the HPV and CST groups with participant categorical and continuous metadata was computed by Fisher’s exact/Chi-square and Mann-Whitney unpaired nonparametric tests, respectively. A p-value of < 0.05 was used as the level of significance. Odds ratios (OR) with corresponding 95% confidence intervals (CI) were used to measure the magnitude of associations.

The alpha diversity indices of the various metadata categories (CST, HPV, HR-HPV, HIV, CD4) were compared using Mann-Whitney unpaired and Kruskal-Wallis nonparametric tests. For beta diversity comparisons, the Adonis nonparametric test with 999 permutations was used. The distance matrix was used to calculate the effect size (R^2^ value) that showed the extent of variation explained by the metadata category. A p-value of < 0.05 was used to measure the statistical significance.

## Supplementary information

**Additional file 1: Table S1.** Top 40 most abundant families in penile microbiota of heterosexually-active Black South African men.

**Additional file 2: Figure S1.** Relative abundances of genera in the 238 men. Only genera that occurred at ≥0.08% relative abundances are shown. Each dot on the x-axis represents a participant. The horizontal (solid) lines separate the genera of the different phyla.

**Additional file 3: Table S2.** Differentially abundant genera in men with *Corynebacterium*-dominated versus diverse penile microbiota.

**Additional file 4: Figure S2.** Alpha and beta diversity measures of penile microbiota. Comparison of the alpha diversity of penile microbiota grouped by a) human papillomavirus (HPV) infection status, and b) human immunodeficiency virus (HIV) and HPV co-infection status. In each plot, the box ranges from the first to the third quartile, with the median represented by the horizontal line. The whiskers extend to the smallest and largest non-outliers and outliers are represented by dots. Comparison of beta diversity (UniFrac distance) of the penile microbiota grouped by c) HPV infection status, and d) HIV and HPV co-infection status. The first two principal coordinate axes of variations and the percentage variation explained by each (Axis.1: 45.3% and Axis.2: 14.9%) are shown. Each solid point is a bacterial community.

**Additional file 5: Figure S3.** Potential biomarkers for high-risk HPV (HR-HPV) infection by LEfSe in men without HIV infection. a) Histogram of differentially abundant taxa in penile microbiota of HIV-negative men with and without HR-HPV infections identified by LEfSe, and b) a six-level cladogram with a taxonomic hierarchical structure. Each coloured solid represents a taxon and its diameter is proportional to the taxon’s relative abundance. Blue and green solids represent statistically significant taxon ranks in HPV-positive and negative group, respectively. Only features with logarithmic LDA scores > 2.0 or < − 2.0 are shown. Asterisks indicate significantly differentially abundant taxa with q < 0.2 after FDR correction.

**Additional file 6.** Metadata associated with all samples used in this study.

**Additional file 7.** Unrarefied operational taxonomic unit table (with genus taxonomic assignments) used for this study.

## Data Availability

The datasets generated and/or analysed during the current study are available in the NCBI Short Read Archive (SRA) under BioProject accession number PRJNA559354 (https://www.ncbi.nlm.nih.gov/sra/PRJNA559354). Metadata and the unrarefied OTU table (with genus taxonomic classifications) have all been included as Additional files 6 and 7, respectively. The mothur modified SOP (mothur_batch.txt), Python (*taxonomy_mothur_abundance_silvaDB_v1.2.py*) and R scripts (*biodiversity_calculator.R* and *betaDiv.R*) used to assess the penile microbiota composition and diversity are available in GitHub (https://github.com/DoHarris/microbiota).

## Abbreviations

16S rRNA: 16-Svedberg ribosomal RNA
β-globin PCR: Beta globin
*Ae*: Aerobic
*An*: Anaerobic
BV: Bacterial vaginosis
CD4: Cluster of differentiation 4
CI: Confidence interval
CST: Community state type
DNA: Deoxyribonucleic acid
dNTP: Deoxyribonucleotide triphosphate
*FAn*: Facultative anaerobic
HIV: Human immunodeficiency virus
HPV: Human papillomavirus
HR-HPV: High-risk human papillomavirus
IQR: Interquartile range
LDA: Linear discriminant analysis
LEfSe: Linear discriminant analysis (LDA) effect size (LEfSe)
LR-HPV: Low-risk human papillomavirus
*MAe*: Microaerophilic
OD: Odds ratio
OTU: Operational taxonomic unit
PCoA: Principal coordinates analysis
PCR: Polymerase chain reaction
RDP: Ribosomal database project
SOP: Standard operating procedure
STI: Sexually transmitted infection
STM: Specimen transport medium
Taq: *Thermus aquaticus*
