# Supplementary material for: The penile microbiota of Black South African men: relationship with human papillomavirus and HIV infection
Source: BMC Microbiol. 2020 Apr 6;20:78. doi: 10.1186/s12866-020-01759-x (PMC7137192; doi:10.1186/s12866-020-01759-x)
Supplement: Supplementary file 1 — Additional file 1: Table S1. Top 40 most abundant families in penile microbiota of heterosexually-active Black South African men. [file 12866_2020_1759_MOESM1_ESM.docx]

Additional file 1: Table S1 Top 40 most abundant families in penile microbiota of heterosexually-active Black South African men

| **Family** | **All participants** | **HIV-negative men (N = 150)^#^** | **HIV-positive men (N = 88)^*^** | **Oxygen requirement** |
| --- | --- | --- | --- | --- |
|  | **% relative abundance** | **% relative abundance (sd)** | **% relative abundance (sd)** |  |
| *Corynebacteriaceae* | 47.19 | 49.71 (28.61) | 42.90 (26.76) | Facultative anaerobic |
| *Prevotellaceae* | 6.56 | 5.58 (11.10) | 8.22 (14.64) | Anaerobic |
| Unclassified *Clostridiales* | 5.61 | 5.21 (11.22) | 6.30 (10.52) | Unidentified |
| *Porphyromonadaceae* | 4.94 | 4.47 (9.83) | 5.73 (10.94) | Anaerobic |
| *Staphylococcaceae* | 4.57 | 3.61 (5.09) | 6.21 (9.50) | Facultative anaerobic |
| *Bifidobacteriaceae* | 3.88 | 4.49 (10.98) | 2.84 (7.66) | Anaerobic/Facultative anaerobic |
| *Lactobacillaceae* | 3.81 | 3.92 (12.48) | 3.64 (14.15) | Microaerophilic/Facultative anaerobic |
| *Veillonellaceae* | 3.31 | 3.06 (4.68) | 3.74 (4.99) | Anaerobic |
| *Moraxellaceae* | 3.12 | 3.02 (8.26) | 3.30 (9.35) | Aerobic |
| *Flavobacteriaceae* | 2.55 | 2.58 (8.05) | 2.51 (6.70) | Aerobic |
| *Clostridiales Incertae Sedis XI* | 2.23 | 2.14 (2.67) | 2.39 (2.90) | Anaerobic |
| *Micrococcaceae* | 1.39 | 1.44 (4.09) | 1.31 (3.70) | Aerobic |
| *Leptotrichiaceae* | 1.13 | 0.97 (3.14) | 1.42 (4.42) | Anaerobic/Facultative anaerobic |
| Unclassified Bacteria | 0.85 | 0.91 (1.03) | 0.74 (1.10) | Unidentified |
| *Brevibacteriaceae* | 0.66 | 0.70 (1.69) | 0.58 (1.00) | Aerobic |
| *Intrasporangiaceae* | 0.60 | 0.82 (3.91) | 0.23 (0.75) | Aerobic |
| *Aerococcaceae* | 0.60 | 0.56 (0.73) | 0.67 (1.09) | Facultative anaerobic |
| Unclassified *Actinomycetales* | 0.57 | 0.65 (0.66) | 0.44 (0.64) | Unidentified |
| *Ruminococcaceae* | 0.55 | 0.57 (1.55) | 0.51 (0.83) | Anaerobic/Facultative anaerobic |
| *Rhodobacteraceae* | 0.55 | 0.46 (1.07) | 0.70 (1.69) | Aerobic |
| *Coriobacteriaceae* | 0.54 | 0.67 (1.92) | 0.33 (0.52) | Anaerobic |
| *Streptococcaceae* | 0.43 | 0.51 (2.75) | 0.30 (0.60) | Facultative anaerobic |
| *Dermabacteraceae* | 0.42 | 0.40 (0.51) | 0.47 (0.52) | Facultative anaerobic |
| *Xanthomonadaceae* | 0.41 | 0.37 (1.35) | 0.47 (1.59) | Aerobic |
| *Lachnospiraceae* | 0.36 | 0.33 (1.31) | 0.41 (0.87) | Anaerobic |
| *Microbacteriaceae* | 0.26 | 0.25 (0.57) | 0.28 (0.54) | Microaerophilic/aerobic |
| *Fusobacteriaceae* | 0.22 | 0.05 (0.33) | 0.52 (2.48) | Anaerobic |
| *Campylobacteraceae* | 0.18 | 0.17 (1.06) | 0.21 (0.67) | Microaerophilic |
| *Propionibacteriaceae* | 0.17 | 0.20 (0.48) | 0.11 (0.39) | Microaerophilic/Facultative anaerobic |
| *Comamonadaceae* | 0.15 | 0.12 (0.75) | 0.21 (1.01) | Aerobic |
| *Pseudomonadaceae* | 0.14 | 0.17 (1.32) | 0.10 (0.24) | Aerobic |
| Unclassified *Bacteroidales* | 0.11 | 0.18 (1.85) | 0.01 (0.02) | Unidentified |
| *Dietziaceae* | 0.11 | 0.14 (0.53) | 0.06 (0.19) | Aerobic |
| Unclassified Proteobacteria | 0.11 | 0.15 (1.67) | 0.04 (0.24) | Unidentified |
| *Neisseriaceae* | 0.11 | 0.09 (0.54) | 0.15 (0.86) | Facultative anaerobic |
| *Actinomycetaceae* | 0.10 | 0.09 (0.31) | 0.11 (0.28) | Anaerobic/facultative anaerobic |
| *Alcaligenaceae* | 0.09 | 0.07 (0.48) | 0.13 (0.83) | Aerobic |
| *Caulobacteraceae* | 0.09 | 0.07 (0.35) | 0.12 (0.43) | Aerobic |
| Unclassified Bacteroidetes | 0.08 | 0.06 (0.51) | 0.12 (0.91) | Unidentified |
| *Enterobacteriaceae* | 0.07 | 0.06 (0.23) | 0.09 (0.32) | Facultative anaerobic |

Abbreviations: sd – standard deviation.

^#^Circumcised men were 92.5% (135/146).

*Circumcised men were 97.6% (80/82).
