# Supplementary figures and images for: The penile microbiota of Black South African men: relationship with human papillomavirus and HIV infection
Source: BMC Microbiol. 2020 Apr 6;20:78. doi: 10.1186/s12866-020-01759-x (PMC7137192; doi:10.1186/s12866-020-01759-x)

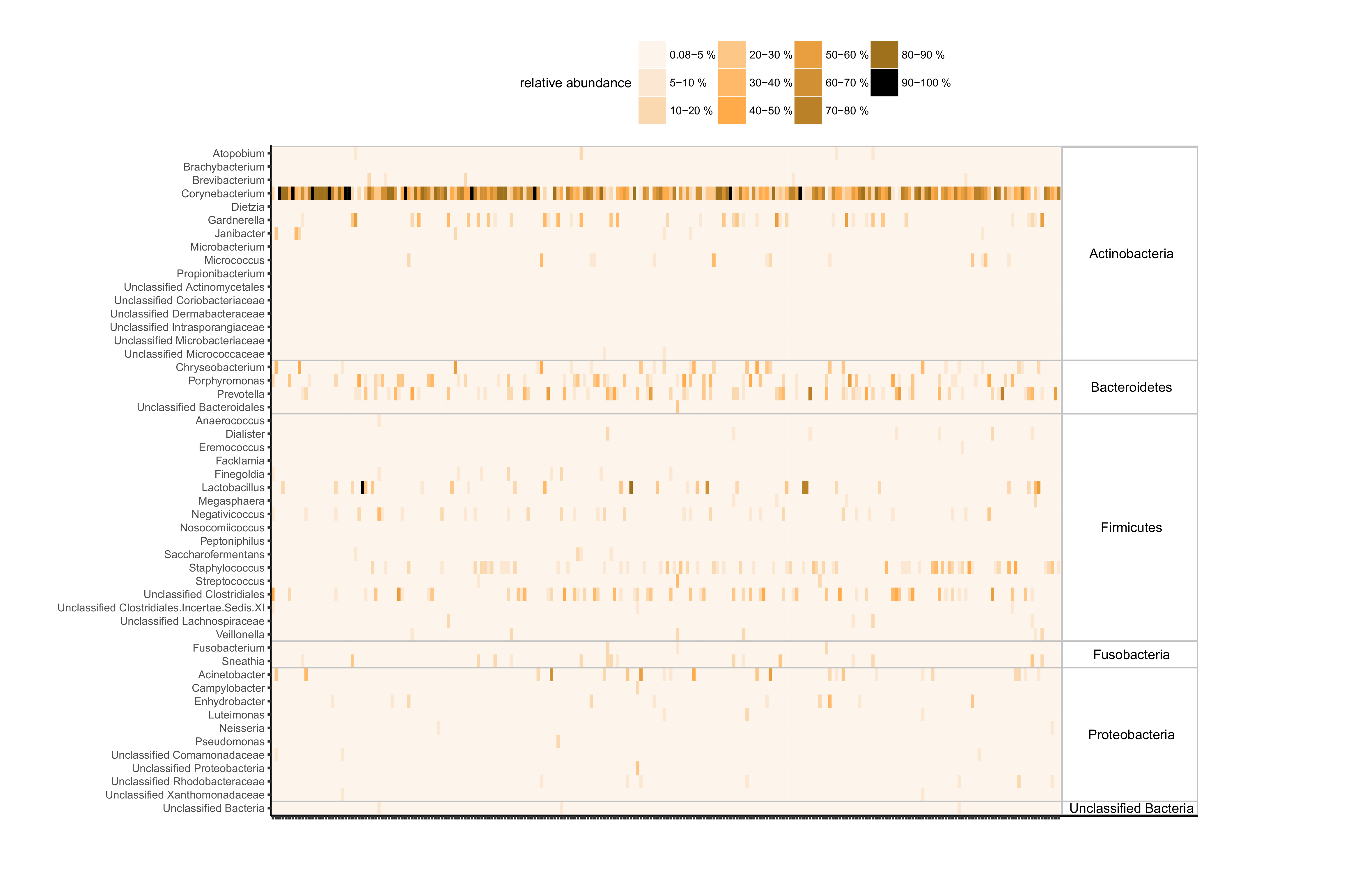

Supplement: Supplementary file 2 — Additional file 2: Figure S1. Relative abundances of genera in the 238 men. Only genera that occurred at ≥0.08% relative abundances are shown. Each dot on the x-axis represents a participant. The horizontal (solid) lines separate the genera of the different phyla. [file 12866_2020_1759_MOESM2_ESM.tif]

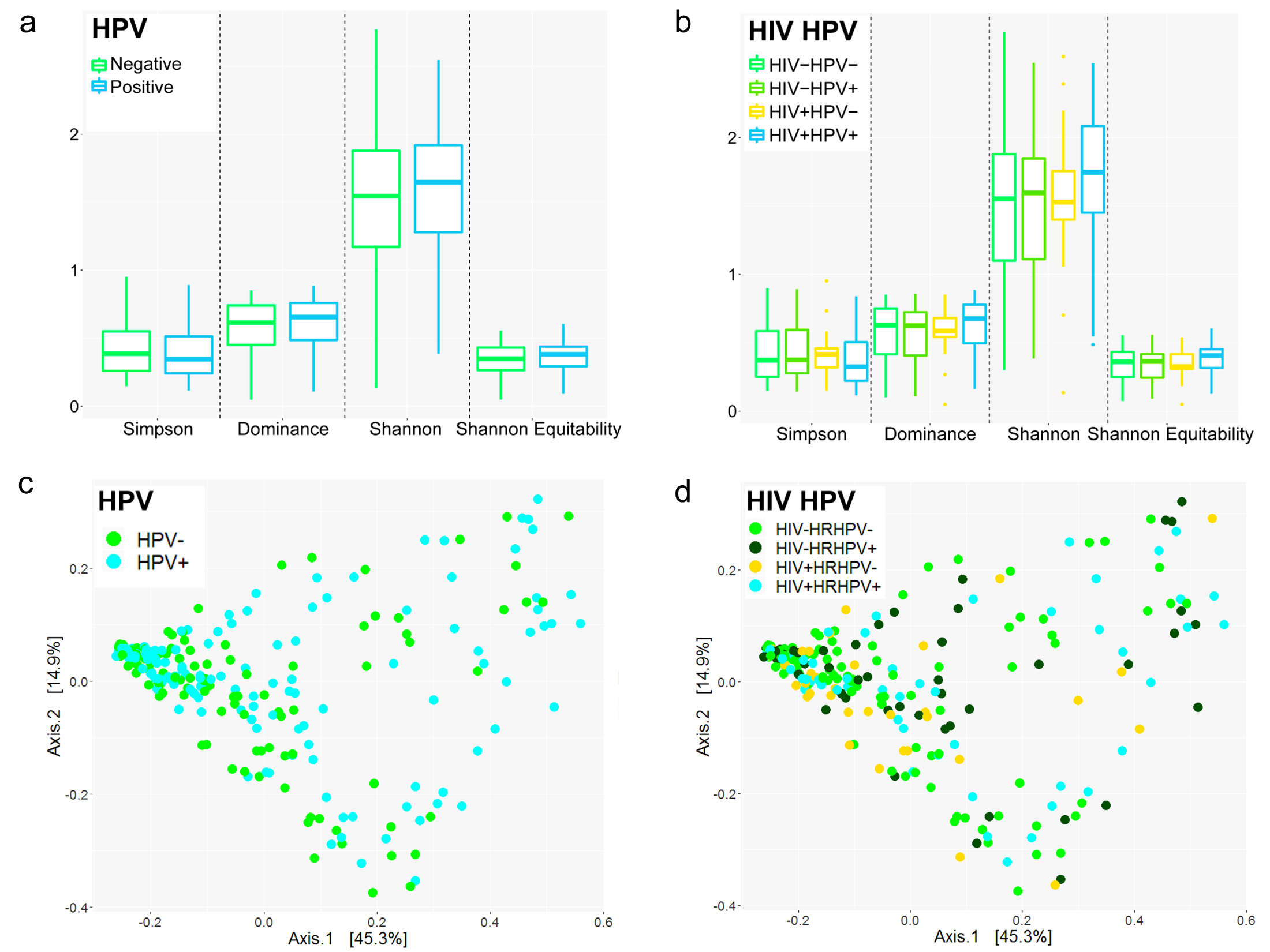

Supplement: Supplementary file 4 — Additional file 4: Figure S2. Alpha and beta diversity measures of penile microbiota. Comparison of the alpha diversity of penile microbiota grouped by a) human papillomavirus (HPV) infection status, and b) human immunodeficiency virus (HIV) and HPV co-infection status. In each plot, the box ranges from the first to the third quartile, with the median represented by the horizontal line. The whiskers extend to the smallest and largest non-outliers and outliers are represented by dots. Comparison of beta diversity (UniFrac distance) of the penile microbiota grouped by c) HPV infection status, and d) HIV and HPV co-infection status. The first two principal coordinate axes of variations and the percentage variation explained by each (Axis.1: 45.3% and Axis.2: 14.9%) are shown. Each solid point is a bacterial community. [file 12866_2020_1759_MOESM4_ESM.tif]

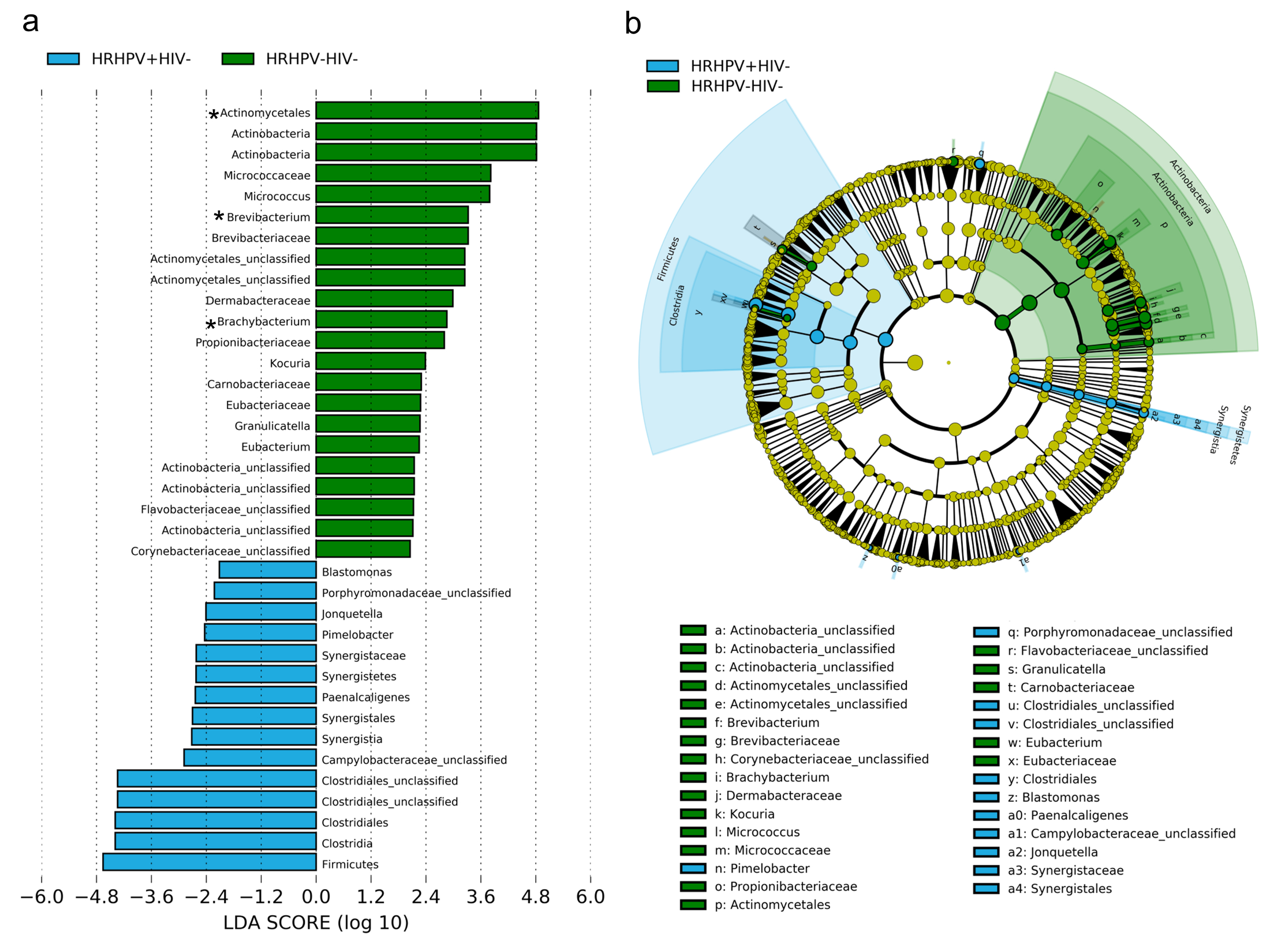

Supplement: Supplementary file 5 — Additional file 5: Figure S3. Potential biomarkers for high-risk HPV (HR-HPV) infection by LEfSe in men without HIV infection. a) Histogram of differentially abundant taxa in penile microbiota of HIV-negative men with and without HR-HPV infections identified by LEfSe, and b) a six-level cladogram with a taxonomic hierarchical structure. Each coloured solid represents a taxon and its diameter is proportional to the taxon’s relative abundance. Blue and green solids represent statistically significant taxon ranks in HPV-positive and negative group, respectively. Only features with logarithmic LDA scores > 2.0 or < − 2.0 are shown. Asterisks indicate significantly differentially abundant taxa with q < 0.2 after FDR correction. [file 12866_2020_1759_MOESM5_ESM.tif]
