# Supplementary material for: The penile microbiota of Black South African men: relationship with human papillomavirus and HIV infection
Source: BMC Microbiol. 2020 Apr 6;20:78. doi: 10.1186/s12866-020-01759-x (PMC7137192; doi:10.1186/s12866-020-01759-x)
Supplement: Supplementary file 3 — Additional file 3: Table S2. Differentially abundant genera in men with Corynebacterium-dominated versus diverse penile microbiota. [file 12866_2020_1759_MOESM3_ESM.docx]

**Additional file 3: Table S2** **Differentially abundant genera in men with *Corynebacterium*-dominated versus diverse penile microbiota**

| ***Corynebacterium*-dominated penile microbiota (CST-1)** | **Diverse penile microbiota (CSTs 2-5)** |
| --- | --- |
| *Corynebacterium*, *Staphylococcus*, *Enhydrobacter*, *Brevibacterium*, unclassified *Bacillales*, unclassified *Bacilli*, *Exiguobacterium*, unclassified *Actinomycetales*,  *Eremococcus*, unclassified *Dermabacteraceae*, *Gp*, *Facklamia*, *Propionibacterium*, unclassified *Planococcaeae*, *Anaerococcus*, unclassified Bacteria, *Neisseria*, unclassified *Oxalobacteraceae*, unclassified *Nocardioidaceae*, unclassified *Sphingomonadaceae*, *Delftia*, *Methylobacterium*, *Naxibacter*, *Zimmermannella*, *Nosocomiicoccus*, *Dermabacter*, *Brachybacterium*, *Gemmiger*, *Clostridium sensu stricto*, *Bifidobacterium*, unclassified *Staphylococcaceae*, *Psychrobacter*, *Roseburia*, *Blautia*, *Jeotgalicoccus*, *Tessaracoccus*, *Salinicoccus*, unclassified *Neisseraceae*, *Sphingomonas*, *Lachnospiraceae incertae sedis* | *Prevotella*, unclassified *Clostridiales*, *Porphyromonas*, *Gardnerella*, *Negativicoccus*, *Sneathia*, *Dialister*, *Finegoldia*, *Saccharofermentans*, unclassified *Clostridiales Incertae Sedis XI*, unclassified *Incertae Sedis XI*, *Atopobium*, *Campylobacter*, *Micrococcus*, unclassified *Bacteroidales*, *Streptococcus*, unclassified Bacteroidetes, *Bacteroides*, *Pseudomonas*, unclassified *Porphyromonadaceae*, *Murdochiella*, unclassified *Micrococcaceae*, *Haemophilus*, *Hallella*, unclassified *Peptostreptococcaceae*, *Peptococcus*, *Parvimonas*, unclassified *Veillonellaceae*, *Sutterella*, unclassified *Coriobacteriaceae*, *Varibaculum*, unclassified *Prevotellaceae*, *Mobiluncus*, *Mycoplasma*, *Citrobacter*, *Aeromonas*, *Brucella*, unclassified Firmicutes, *Howardella*, *Pyramidobacter*, *Olsenella*, *Granulicatella*, unclassified *Aerococcaceae*, *Kocuria*, *Arcanobacterium*, unclassified *Brucellaceae* |

Abbreviations: CST – community state type.

*Lactobacillus*-dominated penile microbiota (CST-6) were excluded from these analyses because they were neither diverse nor dominated by *Corynebacterium*.

Only genera at logarithmic LDA scores >2.0 or <-2.0 (p<0.05, q<2.0) are shown.
